# Five‐year survival and clinical correlates among patients with advanced non‐small cell lung cancer, melanoma and renal cell carcinoma treated with immune check‐point inhibitors in Australian tertiary oncology centres

**DOI:** 10.1002/cam4.5468

**Published:** 2022-11-20

**Authors:** Lauren J Brown, Ines Pires da Silva, Tania Moujaber, Bo Gao, Rina Hui, Howard Gurney, Matteo Carlino, Adnan Nagrial

**Affiliations:** ^1^ Crown Princess Mary Cancer Centre Westmead Hospital Westmead New South Wales Australia; ^2^ Blacktown Cancer and Haematology Centre Blacktown Hospital Blacktown New South Wales Australia; ^3^ University of Sydney Camperdown New South Wales Australia; ^4^ Westmead Institute for Medical Research Westmead New South Wales Australia; ^5^ Melanoma Institute Australia Wollstonecraft New South Wales Australia; ^6^ Macquarie University Health Cancer Services Macquarie University Sydney New South Wales Australia

**Keywords:** immune checkpoint inhibitors, immunotherapy, melanoma, non‐small cell lung cancer, renal cell carcinoma

## Abstract

**Aims:**

There is robust trial evidence for improved overall survival (OS) with immunotherapy in advanced solid organ malignancies. The real‐world long‐term survival data and the predictive variables are not yet known. Our aim was to evaluate factors associated with 3‐year and 5‐year OS for patients treated with immune checkpoint inhibitors (ICIs).

**Methods:**

We performed a retrospective study of patients who received ICIs as management of advanced solid organ malignancies in two tertiary Australian oncology centres from 2012–2017. Data pertaining to clinical characteristics, metastatic disease burden, immune‐related adverse events (IRAEs) and tumour responses were collected and their relationship to survival examined.

**Results:**

In this analysis of 264 patients, 202 (76.5%) had melanoma, 46 (17.4%) had non‐small cell lung cancer (NSCLC), 12 (4.5%) had renal cell carcinoma (RCC) and 4 (1.5%) had mesothelioma. The 5‐year OS rates were 42.1% in patients with melanoma, 19.6% with NSCLC, 75% with RCC, and none of the mesothelioma patients were alive at 5 years.

In multivariate analysis, an ECOG score of 0 (Hazard ratio [HR] 0.39; 95% confidence interval [CI] 0.23–0.66; *p* < 0.001) and the occurrence of IRAE's of any grade (HR 0.61; 95% CI 0.37–0.95; *p* = 0.05) were associated with better 5‐year survival. The presence of bone metastases (HR 1.62; 95% CI 1.03–2.82; *p* = 0.05) and liver metastases (HR 1.76; 95% CI 1.07–2.89; *p* = 0.03) were associated with worse 5‐year survival.

**Conclusions:**

These results support the long‐term benefits of immunotherapy that in some patients, extend to at least 5 years. ECOG performance status of 0 and the occurrence of irAEs are associated with better long‐term survival. Survival is significantly influenced by metastatic site and cancer type. These predictive clinical correlates aid discussions and planning in the delivery of ICIs to patients.

## Graphical abstract

Immunotherapy in solid organ tumours has long term benefits. Patients with ECOG 0 and irAE have an increased likelihood of survival at 5 years. Patients with liver and bone metastases have an increased likelihood of survival at 5 years.

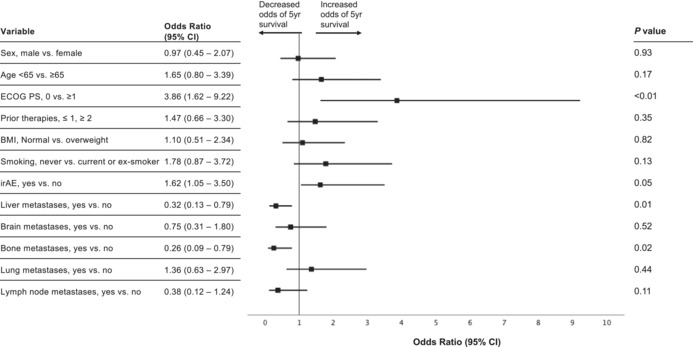

## 1 INTRODUCTION

Indications for the use of immune checkpoint inhibitors (ICIs), without concurrent chemotherapy or targeted therapy, have broadened. ICIs are now approved for use in first or later line therapies in melanoma,^1^, ^2^ renal cell carcinoma (RCC),^3^, ^4^ non‐small cell lung cancer (NSCLC),^5^, ^6^, ^7^, ^8^, ^9^ mesothelioma,^10^, ^11^ hepatocellular carcinoma,^12^ microsatellite instability‐high colorectal carcinoma,^13^ urothelial cancer^14^, ^15^ and head and neck cancers.^16^

There are now published trials demonstrating the long‐term survival rates for patients with metastatic cancer receiving immunotherapy. For melanoma, the published 5‐year OS rates for patients treated in the first line with nivolumab and ipilimumab vs. nivolumab vs. ipilimumab were 52% vs. 44% vs. 26% respectively.^2^ For NSCLC, published 5‐year OS rates for programmed cell death protein‐1 (PD1) inhibitors, or anti‐PD1 alone are as high as 32% in the first line^5^ and between 13%^9^ and 25%^7^ in the second line. For RCC, the 5‐year OS rates with second‐line anti‐PD1 alone are 26%,^17^ and 48% with first‐line combination anti‐PD1 and ipilimumab.^18^ It is clear that ICIs demonstrate impressive incremental enduring survival benefit for select cancer patients. However, yet to be reported outside the clinical trial setting are real‐world survival data and the identification of inherent unique clinical predictors. These data are needed to better inform prognostication and treatment discussions.

As such, we assessed long‐term survival outcomes among patients with advanced melanoma, NSCLC, RCC and mesothelioma in two Australian tertiary oncology centres. We sought to identify baseline and on‐treatment factors associated with long‐term OS by examining baseline clinical characteristics, adverse events and tumour response.

## 2 METHODS

Following approval of our local institutional review board, we retrospectively identified patients who were treated with immunotherapy, including PD1 inhibitors, or anti‐PD1 (nivolumab, pembrolizumab), cytotoxic T‐lymphocyte associated protein 4 (CTLA4) inhibitors (ipilimumab) or combination immunotherapy. Patients were included if they received treatment between September 2012–May 2017, allowing five‐year outcomes to be evaluated. The inclusion criteria were patients with metastatic (stage 4) solid organ tumours treated with ≥1 dose of immunotherapy and had at least one tumour progression assessment, either via a computed tomography (CT) scan or fluorodeoxyglucose‐positron emission tomography (FDG‐PET) imaging. Patients with unresectable stage III melanoma and stage III NSCLC, or those unable to be managed with surgical resection or definitive chemoradiation were excluded.

### 2.1 Study design and treatment

Pertinent data were extracted from institutional electronic medical records. Baseline patient demographics were collected including age, gender, Eastern Cooperative Group (ECOG) Performance Status, body mass index (BMI), smoking status, lines and types of prior therapy. Data regarding the use of concurrent antibiotics were unavailable. The number and anatomic location of metastatic sites were recorded. Immune‐related adverse events (irAEs) were recorded and the corresponding clinical diagnosis and grade defined by the Common Terminology Criteria for Adverse Events (CTCAE) criteria version 5.0.^19^ Response was assessed using Response Evaluation Criteria in Solid Tumours (RECIST) 1.1^20^ and iRECIST guidelines^21^ and/or FDG‐PET. As per local institutional practice, patients underwent progress imaging every 3–4 months in the first 2 years after commencing treatment. If patients had responses beyond 24 months, imaging was performed every 6 months.

### 2.2 Endpoints

The endpoints of this study were: Overall response rate (ORR), defined as the proportion of patients who had a partial or complete response to treatment; progression free survival (PFS), defined as time from starting anti‐PD1 or ipilimumab to disease progression or death or last follow‐up; OS, defined as time from starting anti‐PD1 or ipilimumab to death or last follow‐up; and safety, defined as proportion of patients with immune‐related adverse events. Where patients had been previously treated with ipilimumab, the start of the second‐line anti‐PD1 +/‐ipilimumab therapy was used as the commencement for PFS and OS.

### 2.3 Statistical analysis

Categorical and continuous variables are summarised using percentages and medians. Time to event measures, including OS and PFS were calculated using the Kaplan–Meier method and compared using the log‐rank test. All patients were censored at last available follow‐up. Univariate and multivariate Cox regression analyses served to determine variables associated with each of the endpoints. Univariate and multivariate analyses were performed to assess the associations between baseline clinical data (at the time of commencement of immunotherapy), and outcomes, namely ORR, 5‐year PFS and landmark 3‐year and 5‐year OS. The estimated hazard ratio (HR) and its 95% confidence interval (CI) were used to assess the strength of these associations. Odds ratios (OR) and 95% CI based on logistic regression were also provided. For ORR, exact binomial 95% CIs were determined using Clopper‐Pearson methods. Two‐sided significance levels (nominal threshold of 0.05) were reported, and IBM SPSS software, version 27, was used for statistical analysis. The reported analyses, including OS, were exploratory in nature. Final data analysis was performed on 25th June 2022.

## 3 RESULTS

### 3.1 Patient characteristics

**FIGURE 1:**
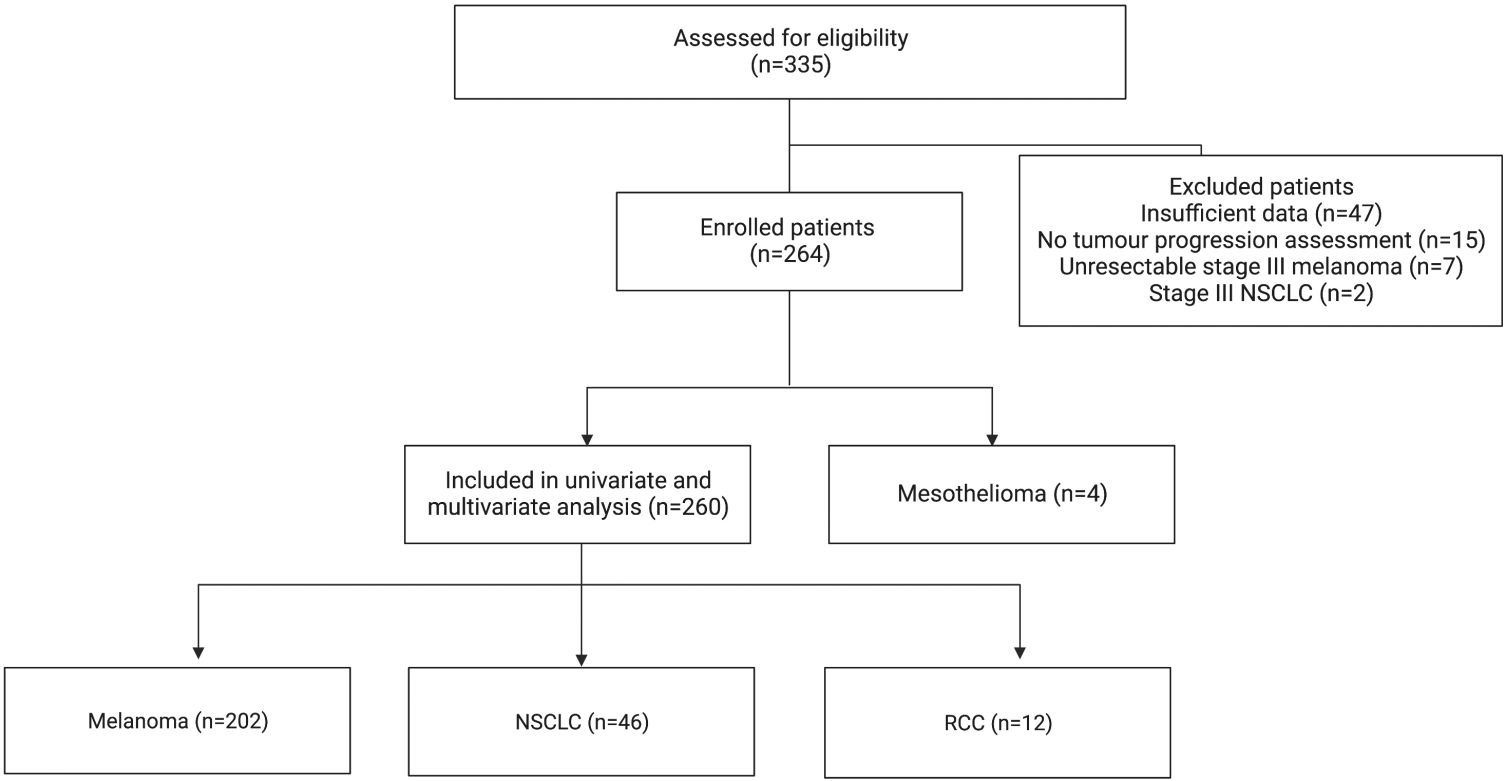
Flow diagram for inclusion and exclusion of patients. n, number; NSCLC, non‐small cell lung cancer; RCC, renal cell carcinoma.

We identified 335 consecutive eligible patients who received immunotherapy. Of these, a total of 264 patients had metastatic (stage 4) cancer with available data and response imaging for evaluation (Figure 1). Sixty‐one patients (23.1%) were treated with immunotherapy on clinical trials. 202 (76.5%) had melanoma (137 [67.8%] male; median age, 65 years [range, 20–93 years]), 46 (17.4%) had NSCLC (25 [54.3%] male; median age, 67 years [range, 35–84 years]), 12 (4.5%) had RCC (11 [91.7%] male; median age, 61 years [range, 38–83 years]), and 4 (1.5%) had mesothelioma (3 [75%] male; median age, 71 years [range, 61–77 years]). Univariate and multivariate assessments were limited to 260 patients, excluding the patients with mesothelioma, given the small numbers of patients and limited survival of this subgroup, likely driven by tumour factors rather than clinical correlates.

Baseline patient characteristics by disease type are summarised in Table 1. Of the patients with melanoma, 125 (61.9%) were treatment‐naïve, 78 (38.6%) had an elevated LDH at commencement of immunotherapy, 39 (19.3%) were BRAF V600E positive, 150 (74.3%) were Stage VI M1C/D by the AJCC Melanoma Staging 8th Edition.^22^ Of the patients with NSCLC, 7 (15.2%) were treatment‐naïve. PDL1 expression status was not available for the majority of patients, given this was not present in mainstream clinical practice prior to 2017. For the patients with RCC, 9 (75%) were treatment‐naïve and all were clear cell histologic subtype and had an intermediate International Metastatic RCC Database Consortium (IMDC) Risk Score.^23^

**TABLE 1:** Baseline characteristics

Demographics	*N* (%)	Melanoma *n* = 202 (77%)	NSCLC *n* = 46 (17%)	RCC *n* = 12 (4.5%)	Mesothelioma *n* = 4 (1.5%)
Age, median (range), y	66 (20–93)	65 (20–93)	67 (35–84)	61 (38–83)	71 (61–77)
Gender					
Male	176 (67)	137 (68)	25 (54)	11 (92)	3 (75)
Female	88 (33)	65 (32)	21 (46)	1 (8)	1 (25)
ECOG PS 0	175 (66)	142 (70)	21 (46)	10 (83)	2 (50)
Therapy					
Single agent anti‐PD1	197 (75)	149 (74)	41 (89)	3 (25)	4 (100)
Single agent IPI	23 (9)	23 (11)	0 (0)	0 (0)	0 (0)
Combination anti‐PD1 + IPI	44 (17)	30 (15)	5 (11)	9 (75)	0 (0)
Line of systemic therapy					
1st	141 (54)	125 (62)	7 (15)	9 (75)	0 (0)
2nd	104 (39)	64 (32)	35 (76)	2 (17)	3 (75)
3rd	19 (7)	13 (6)	4 (9)	1 (8)	1 (25)
LDH					
Elevated		78 (39)			
Normal		110 (54)			
Unknown		14 (7)			
Stage (AJCC 8th Edition)					
M1A/B		52 (26)			
M1C/D		150 (74)			
IMDC score					
Poor risk				0 (0)	
Intermediate risk				12 (100)	
Favourable risk				0 (0)	
Metastatic sites of disease^a^					
Bone	39 (14)	24 (12)	12 (26)	3 (25)	0 (0)
Liver	75 (28)	63 (31)	10 (22)	2 (17)	0 (0)
Lung	179 (64)	116 (57)	43 (93)	7 (58)	1 (25)
Brain	62 (23)	54 (27)	8 (17)	0 (0)	0 (0)
Lymph node	225 (85)	168 (83)	45 (98)	11 (92)	1 (25)
Serosal	29 (11)	12 (6)	12 (26)	2 (17)	3 (75)
BMI					
Underweight	15 (6)	4 (2)	9 (20)	0 (0)	2 (50)
Normal	88 (33)	62 (31)	19 (41)	6 (50)	1 (25)
Overweight	38 (14)	28 (14)	8 (17)	2 (17)	0 (0)
Obese	27 (10)	19 (9)	4 (8)	3 (25)	1 (25)
Severely obese	1 (<1)	0 (0)	0 (0)	1 (8)	0 (0)
Not known	95 (36)	89 (44)	6 (13)	0 (0)	0 (0)
Smoking status					
Smoker	15 (6)	6 (3)	9 (20)	0 (0)	0 (0)
Ex‐smoker	111 (42)	78 (39)	26 (56)	5 (42)	2 (50)
Never‐smoker	128 (48)	108 (53)	11 (24)	7 (58)	2 (50)
Not Known	10 (4)	10 (5)	0 (0)	0 (0)	0 (0)

Abbreviations: AJCC, American Joint committee on Cancer; BMI, Body mass index; ECOG PS, Eastern Cooperative Oncology Group Performance Status; IMDC, International Metastatic RCC Database Consortium; IPI, ipilimumab; N, Number; NSCLC, non‐small cell lung cancer; LDH, lactate dehydrogenase; PD1, programmed cell death protein‐1; RCC, renal cell carcinoma; y, Years.

^a^ Some included patients have more than one site of metastatic disease.

An ECOG performance status of 0 was reported in 175 (65.5%) patients. Specifically, 142 (70.3%) with melanoma, 21 (45.7%) with NSCLC, 10 (83.3%) with RCC and 2 (50%) with mesothelioma.

Anti‐PD1 monotherapy was used in 197 (74.6%) patients, ipilimumab in 23 (8.7%) patients and combination therapy in 44 (16.6%) patients, followed by maintenance anti‐PD1 monotherapy in 41 patients (Table S1). The median number of cycles received was 9 (range 2–156). Prior therapy for metastatic disease occurred in 123 (47%) patients. Prior ipilimumab treatment was given in 33 (27%) of these, all of whom had melanoma.

At the time of the analysis, the minimum follow up for the cohort was 60 months and 100 (37.9%) patients were alive, of whom 86 (42.6%) had melanoma, 7 (15.2%) had NSCLC and 7 (58.3%) had RCC.

### 3.2 Objective response

The ORR (complete and partial response) to ICIs was 51.1% for all tumour types (135 of 264 patients) (Table 2). The ORR was 56.4% for patients with melanoma (114 of 202 patients), 30.4% for patients with NSCLC (14 of 46 patients), 50.0% for patients with RCC (6 of 12 patients) and 25.0% for patients with mesothelioma (1 of 4 patients).

**TABLE 2:** Responses to treatment

	Patients (%)				
Response Assessments^a^	All (*n* = 264)	Melanoma (*n* = 202)	NSCLC (*n* = 46)	RCC (*n* = 12)	Mesothelioma (*n* = 4)
Objective response^b^	135 (51.1)	114 (56.4)	14 (30.4)	6 (50.0)	1 (25.0)
95% CI for response rate, %	45.1–57.2	49.6–63.2	17.1–43.6	21.7–78.3	1.7–67.4
Best overall response					
Complete Response	55 (20.8)	52 (25.7)	2 (4.3)	1 (8.3)	0 (0)
Partial Response	80 (30.3)	62 (30.7)	12 (26.1)	5 (41.7)	1 (25.0)
Stable disease	45 (17.0)	27 (13.4)	12 (26.1)	6 (50.0)	0 (0)
Progressive disease	84 (31.8)	61 (30.2)	20 (43.5)	0 (0)	3 (75.0)
Disease control rate^c^	171 (64.8)	136 (67.3)	25 (54.3)	9 (75.0)	1 (25.0)

Abbreviations: CI, confidence interval; *n*, number; NSCLC, non‐small cell lung cancer; RCC, renal cell carcinoma.

^a^ Response assessments are defined by modified RECIST, version 1.1 or by FDG‐PET imaging modality.

^b^ Complete response and partial response.

^c^ Complete response, partial response, and stable disease for 6 months.

The disease control rate (DCR; complete response, partial response and stable disease ‐ lasting 6 months or more) was 64.8% for all tumour types (171 of 264 patients). The DCR was 67.3% for melanoma patients (136 of 202 patients), 54.3% for patients with NSCLC (25 of 46 patients), 75.0% for patients with RCC (9 of 12 patients) and 25.0% for patients with mesothelioma (1 of 4 patients).

In univariate analysis, performed to assess the association of the clinical variables to ORR (Table S2), age < 65 years (HR 0.65, 95% CI 0.45–0.93, *p* = 0.04), ECOG performance status of 0 (HR 0.34, 95% CI 0.24–0.48, *p* < 0.001), occurrence of irAEs (HR 0.30, 95% CI, 0.20–0.45, *p* < 0.001) and treatment naïve patients (HR 0.41, 95% CI 0.29–0.59, *p* < 0.001) were associated with an increased likelihood of achieving a partial or complete response to immunotherapy. The presence of liver metastases at baseline (HR 1.53, 95% CI 1.05–2.21, *p* = 0.03) was associated with a reduced likelihood of achieving a response.

Further multivariate analysis demonstrated ECOG performance status of 0 (HR 0.41, 95% 0.23–0.73, *p* < 0.01) and occurrence of irAE (HR 0.37, 95% 0.20–0.67, *p* < 0.001) were associated with an increased likelihood of response. The presence of brain metastases at baseline was associated with a reduced likelihood of response (HR 1.83, 95% CI 1.02–3.29, *p* = 0.04).

### 3.3 Progression free survival

**FIGURE 2:**
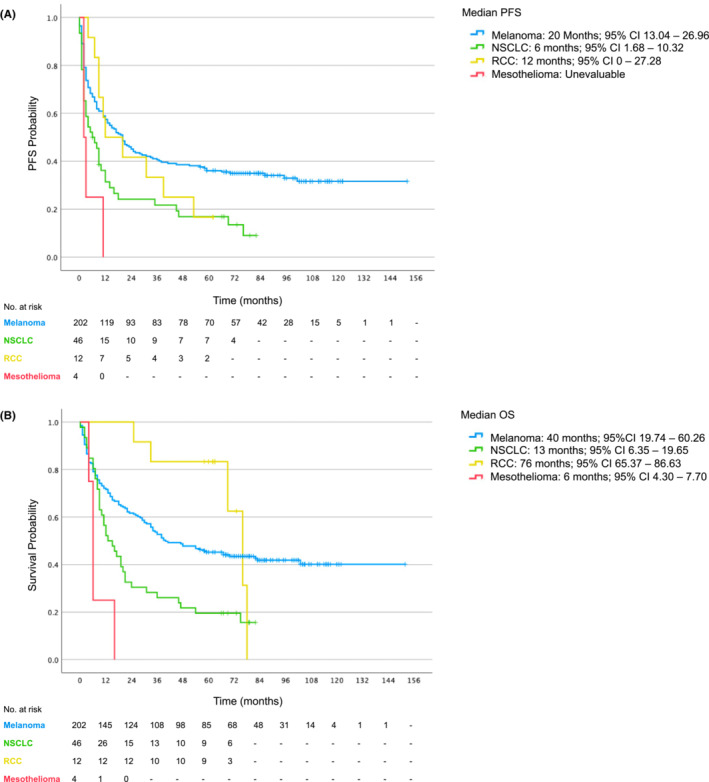
(A) Kaplan Meier curve for progression free survival. (B) Kaplan Meier curve for overall survival. CI, confidence interval; No., number; NSCLC, non‐small cell lung cancer; OS, overall survival; PFS, progression free survival; RCC, renal cell carcinoma.

The median PFS (Figure 2A; Table 3) was 14 months for all patients (95% CI 9.37–18.63). The median PFS for patients with melanoma was 20 months (95% CI 13.04–26.96), NSCLC was 6 months (95% CI 1.68–10.32) and for RCC was 12 months (95% CI 0–27.28). The median PFS for patients with mesothelioma was unable to be evaluated.

**TABLE 3:** Progression free survival and overall survival by tumour type

	Patients				
Survival	All Patients (*n* = 264)	Melanoma (*n* = 202)	NSCLC (*n* = 46)	RCC (*n* = 12)	Mesothelioma (*n* = 4)
Median PFS (months)	14	20	6	12	NE
Median OS (months)	35	40	13	76	6
3 year OS (%)	49.6	53.5	28.3	83.3	0
5 year OS (%)	39.0	42.1	19.6	75.0	0

Abbreviations: CI, confidence interval; *n*, number; NE, not evaluable; NSCLC, non‐small cell lung cancer; OS, overall survival; PFS, progression free survival; RCC, renal cell carcinoma.

At 5 years, the PFS rate for the cohort was 29.9%. The PFS rate at 5 years was 34.7% in patients with melanoma, 15.2% in patients with NSCLC, 16.7% in patients with RCC and 0% in patients with mesothelioma (Table 3).

In univariate analysis (Table S3), age < 65 years (HR 0.74, 95% CI 0.55–1.00, *p* = 0.05), ECOG performance status of 0 (HR 0.42, 95% CI 0.31–0.56, *p* < 0.001), occurrence of irAE (HR 0.46, 95% CI 0.34–0.63, *p* < 0.001) and patients who were treatment naïve (HR 0.53, 95% CI 0.40–0.72, *p* < 0.001) were associated with an increased likelihood of PFS at 5 years. The presence of bone metastases (HR 1.66; 95% CI 1.14–2.40; *p* < 0.01), liver metastases (HR 1.57; 95% CI 1.15–2.14; *p* < 0.01) and lymph node metastases (HR 1.62; 95% CI 1.01–2.57; *p* = 0.04) at baseline had a reduced likelihood of PFS at 5 years.

In multivariate analysis, ECOG performance status of 0 (HR 0.53; 95% CI 0.33–0.84; p < 0.01) was associated with an increased likelihood of PFS at 5 years.

### 3.4 Overall survival

The median OS (Figure 2B; Table 3) for the cohort was 35 months (95% CI 23.82–46.18). The median OS for patients with melanoma was 40 months (95% CI 19.74–60.26), NSCLC was 13 months (95% CI 6.35–19.65), RCC was 76 months (95% CI 65.37–86.63) and with mesothelioma was 6 months (95% CI 4.30–7.70).

The landmark 3‐year OS rate for the cohort was 49.6%. 3‐year OS was 53.5% among patients with melanoma, 28.3% among patients with NSCLC and 83.3% among patients with RCC and 0% in patients with mesothelioma.

The landmark 5‐year OS rate for the cohort was 39.0%. 5‐year OS was 42.1% among patients with melanoma, 19.6% among patients with NSCLC and 75% among patients with RCC (Table 3). Of the 85 patients with melanoma alive at 5 years, 62 (72.9%) were treatment naïve and 23 (27.1%) received prior lines of treatment. Of the 9 patients with NSCLC, 2 (22.2%) were treatment naïve and 7 (77.8%) received prior lines of treatment. For the 9 patients with RCC, 6 (66.7%) were treatment naïve and 3 (33.3%) received prior lines of treatment.

In general, treatment‐naïve patients had a longer median OS with immunotherapy vs. those who received immunotherapy as second or third line treatments. The median OS for patients with melanoma treated with first‐line immunotherapy was not reached, and 27 months for those receiving second or third line immunotherapy (95% CI 9.91–44.10) (Figure S1). The median OS for patients with NSCLC treated with first‐line immunotherapy was 21 months (95% CI 15.87–26.13), and 12 months (95% 8.34–15.67) for those on subsequent lines of immunotherapy The median OS for patients with RCC treated with first‐line immunotherapy was 69 months (95% CI 46.72–73.50), whereas in subsequent lines the median OS was longer at 76 months (95% CI 65.37–86.63).

In univariate analysis (Table 4), age < 65 years (HR 0.70; 95% CI 0.51–0.96; *p* = 0.03), ECOG performance status of 0 (HR 0.34; 95% CI 0.25–0.47; *p* < 0.001), the occurrence of irAEs (HR 0.42; 95% CI 0.30–0.59; <0.001) and patients who were treatment naïve (HR 0.51; 95% CI 0.37–0.70; *p* < 0.001) had an increased 5‐year survival. The presence of bone (HR 1.88; 95% CI 1.28–2.78; *p* < 0.01) and liver (HR 1.74; 95% CI 1.25–2.41; *p* < 0.001) metastases at baseline were associated with reduced 5‐year survival.

**TABLE 4:** Univariate and multivariable analysis for survival at 5 years with hazard ratio

			Univariate analysis		Multivariate analysis	
Variable	Patients (*n* = 260^a^)	5‐year OS, %	hazard ratio (95% CI)	*p*‐value	hazard ratio (95% CI)	*p*‐value
Age, y					0.76 (0.46–1.25)	
< 65	119	46.6	0.70 (0.51–0.96)			
≥ 65	141	39.8		0.03		0.28
Sex						
Male	173	38.2	1.12 (0.80–1.57)		1.10 (0.67–1.79)	
Female	87	43		0.51		0.72
ECOG PS						
0	173	51.7	0.34 (0.25–0.47)		0.39 (0.23–0.66)	
≥1	87	16.1		<0.001		<0.001
BMI						
Normal/underweight	102^b^	48.0	1.46 (0.92–2.32)		1.18 (0.72–1.93)	
Overweight	65^b^	58.5		0.11		0.51
Smoking status						
Never smoker	126^b^	42.9	0.85 (0.62–1.17)		0.84 (0.51–1.38)	
Current/prior smoker	124^b^	37.4		0.31		0.49
irAE						
Yes	109	55	0.42 (0.30–0.59)			
No	151	28.7		<0.001	0.61 (0.37–0.95)	0.05
Prior systemic therapies						
0	141	49.6	0.51 (0.37–0.70)		0.76 (0.44–1.32)	
≥1	119	27.7		<0.001		0.33
Bone metastases						
Yes	39	18.4	1.88 (1.28–2.78)		1.62 (1.03–2.82)	
No	221	43.4		<0.01		0.05
Liver metastases						
Yes	75	25.3	1.74 (1.25–2.41)		1.76 (1.07–2.89)	
No	185	45.7		<0.001		0.03
Lung metastases						
Yes	166	41.2	0.95 (0.69–1.31)		0.98 (0.59–1.64)	
No	94	37.2		0.76		0.95
LN metastases						
Yes	224	37.2	1.64 (0.98–2.75)		2.02 (0.83–4.89)	
No	36	55.6		0.06		0.12
Brain metastases						
Yes	62	38.7	1.13 (0.78–1.62)		1.28 (0.74–2.21)	
No	198	40.1		0.53		0.38

Abbreviations: BMI, body mass index; CI, confidence interval; ECOG PS, European Cooperative Oncology Group Performance Status; HR, hazard ratio; irAE, immune‐related adverse events; LN, lymph nodes; *n*, number; OS, overall survival.

^a^ Excludes patients with mesothelioma (*n* = 4).

^b^ Data missing for some patients.

Further multivariate analysis demonstrated an ECOG of 0 vs. ≥ 1 (HR 0.39; 95% CI 0.23–0.66; p < 0.001) and the occurrence of IRAE's of any grade (HR 0.61; 95% CI 0.37–0.95; *p* = 0.05), was associated with increased 5‐year survival. The presence of bone (HR 1.62; 95% 1.03–2.82; *p* = 0.05) and liver (HR 1.76; 95% 1.07–2.89; *p* = 0.03) metastases at baseline were associated with reduced 5‐year survival.

Following univariate and multivariate analyses, gender, BMI, smoking status, number of prior therapies and presence of lung, brain or lymph node metastases did not significantly influence 5‐year OS.

In univariate analysis performed for 3‐year OS, influential variables were consistent for 3 and 5‐year survival (Table S4). Variables associated with increased likelihood of 3‐year survival were age, ECOG, irAE and patients who were treatment naïve. Variables associated with reduced likelihood of 3‐year survival were presence of bone and liver metastases at baseline as well as high BMI and presence of lymph node metastases at baseline. In multivariate analysis, ECOG and irAE were associated with improved 3‐year OS, and no variables were associated with reduced 3year OS.

Associations of the baseline demographic and clinical data were also assessed using logistic regression models and ORs (Figure 3; Table S5). Kaplan Meier curves for each of the clinical correlates are represented in Figures S2–S13.

**FIGURE 3:**
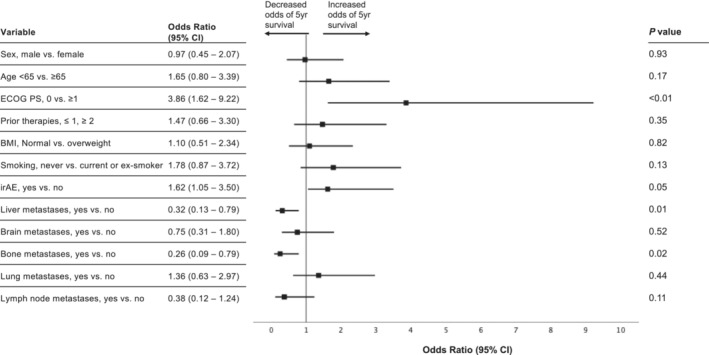
Associations of baseline demographic and clinical characteristics with overall survival at 5 years. BMI, body mass index; CI, confidence interval; ECOG PS, European Cooperative Oncology Group Performance Status; irAE: immune‐related adverse events.

DCR (CR/PR/SD vs. PD) was associated with increased 3‐year OS (HR 0.10; 95% CI 0.06–0.15; *p* < 0.001) and 5‐year OS (HR 0.05; 95% 0.03–0.07; *p* < 0.001).

### 3.5 Melanoma cohort subanalysis

Given the large number of melanoma patients included within the cohort, further subgroup analysis of the melanoma cohort was performed. The median PFS for treatment naïve patients was 31 months (95% CI 5.72–56.28) and 7 months for patients treated with immunotherapy in subsequent lines (95% CI 3.70–10.31) (Figure S14). Median PFS for patients treated with anti‐PD1 and ipilimumab was 37 months (95% CI 0–84.65 months), 18 months (95% CI 12.02–23.98) for anti‐PD1 monotherapy, and 17 months (95% CI 0–43.61) for ipilimumab monotherapy (Figure S15). Median PFS for BRAF V600E positive patients was 5 months (95% CI 1.36–8.64) and 24 months (95% 11.49–36.51) for BRAF V600E negative patients (Figure S16).

Within the melanoma cohort, the median OS was not reached for patients treated with anti‐PD1 and ipilimumab. Median OS was 35 months (95% CI 21.59–48.41) for patients treated with anti‐PD1 monotherapy, and 48 months (95% CI 7.31–88.69) for patients treated with ipilimumab monotherapy (Figure S17). The 3‐year OS was 70.0% for patients treated with anti‐PD1 and ipilimumab, 57.8% with anti‐PD1 monotherapy, and 56.5% with ipilimumab monotherapy. The 5‐year OS was 53.3% with anti‐PD1 and ipilimumab, 40.9% with anti‐PD1 monotherapy, and 34.8% with ipilimumab monotherapy.

The median OS for BRAF V600E positive patients was 13 months (95% CI 0–33.80) and 67 months (95% CI 21.26–112.74) for BRAF V600E negative patients (Figure S18). The 3‐year OS was 33.3% for BRAF V600E positive patients and 58.3% for BRAF V600E negative patients. The 5‐year OS was 47.2% for BRAF V600E positive patients and 28.2% for BRAF V600E negative patients.

Univariate analysis was performed to assess the associations with 5‐year OS in the melanoma cohort (Table S6). Patients with an ECOG of 0 (HR 0.35; 95% CI 0.24–0.51; *p* < 0.001), BRAF V600E negative disease (HR 0.45; 95% CI 0.30–1.68; *p* < 0.001), occurrence of irAE (HR 0.39; 95% CI 0.26–0.58; *p* < 0.001) and treatment naïve patients (HR 0.55; 95% CI 0.38–0.80; *p* < 0.01) had increased likelihood of survival at 5 years. The presence of bone (HR 1.72; 95% CI 1.04–2.84, *p* = 0.04) and liver metastases (HR 1.79; 95% CI 1.23–2.61; *p* < 0.01) at baseline were associated with reduced survival at 5 years in this cohort. Further multivariate analysis demonstrated than an ECOG performance status of 0 (HR 0.40; 95%CI 0.18–0.89; *p* = 0.03) and occurrence of irAE (HR 0.46; 95% CI 0.24–0.91; *p* = 0.03) were associated with increased likelihood of 5‐year survival. The presence of liver metastases (HR 2.74; 95% CI 1.33–5.61; *p* < 0.01) at baseline was associated with reduced 5‐year survival.

### 3.6 Adverse events

Of the 264 patients, irAEs of any grade were reported in 109 (41.2%). The rates of irAE were 70.5% in patients who received combination immunotherapy (31 of 44), 35.5% who received anti‐PD1 monotherapy (70 of 197) and 34.8% that received ipilimumab (8 of 23). This included irAEs in 45.0% of the melanoma cohort (91 of 202), 23.9% of the NSCLC cohort (11 of 46) and 58.3% of the RCC cohort (7 of 12). No patients with mesothelioma experienced irAEs. irAEs of grade 3 or more were experienced by 14.0% of patients (37 of 264). irAEs affecting one or more organ were experienced by 32 patients (12.1%). Immunotherapy was ceased in 74 patients (28.0%) due to an irAE. The most common adverse events were rash (27%), thyroiditis (26%) and hypophysitis (18%). Most irAEs (79.8%) occurred within the first 6 months of immunotherapy. The other types of irAE are summarised in Table S7. As described, occurrence of an irAE was associated with better OS.

## 4 DISCUSSION

Presently unreported, outside clinical trial settings, are real‐world long‐term survival data for patients receiving immunotherapy for late stage cancer, as well as possible clinical predictors of both response to therapy and survival. In our study, we discovered several important findings. Firstly, our survival rates are overtly consistent with those published in pivotal trials of respective cancer types. Secondly, we identified multiple clinical and anatomic variables of significant predictive and prognostic importance. Specifically, ECOG performance status of 0 and the occurrence of irAE were associated with increased likelihood of achieving a partial or complete response and long‐term OS. ECOG performance status of 0 was also associated with increased PFS at 5 years. The presence of brain metastases at baseline was associated with a reduced likelihood of response. The presence of liver and bone metastases were associated with reduced 5‐year OS. We report, to our knowledge, the longest combined clinical follow‐up data for real‐world patients receiving any immunotherapy for the treatment of different cancer types.

For metastatic melanoma, NSCLC without driver mutations, and intermediate‐ or poor‐risk RCC, immunotherapy is now considered standard of care first‐line treatment. Our data are largely consistent with OS rates reported in pivotal trials.

Specifically, in our study, patients with melanoma had 3‐year and 5‐year survival rates of 54.6% and 42.1% respectively. This is comparable to prior large pivotal studies, namely Checkmate 067^2^, ^24^ (combination immunotherapy vs. nivolumab vs. ipilimumab) and Keynote‐006^25^ (pembrolizumab vs. ipilimumab) in metastatic melanoma. Although our finding of better survival in patients with BRAF V600E negative vs. positive disease is discordant with the results of the Checkmate 067 trial^2^ (where patients with BRAF mutations had longer OS), there is evidence that ICIs, when given as second line treatment, have reduced efficacy in the setting of poorer performance status, large disease volume and biological changes.^26^, ^27^, ^28^ The patients in our cohort with BRAF‐mutant melanoma were heavily pre‐treated, all with BRAF inhibitors and some with prior chemotherapy or ipilimumab. It is likely this contributed to poorer outcomes of this subgroup in our cohort. It has subsequently become standard of care that patients with BRAF mutations be treated with immunotherapy up‐front.^2^

In our study, the 3‐year and 5‐year survival rates for NSCLC were 28.3% and 19.5%, respectively. This is similar to the published outcomes of the first and second line studies, the Checkmate 017 and 057 trials^9^, ^29^ (second line nivolumab, received by a majority of our patients), Keynote‐010 trial^7^, ^30^ (second line pembrolizumab) and the Keynote‐024 trial^5^, ^31^ (first line pembrolizumab for tumours with ≥50% PDL1 expression).

For our cohort of patients with RCC, the 3‐year and 5‐year OS rates were 83.3% and 75% respectively. These results stand up to the reported outcomes in the Checkmate‐214 trial^4^, ^32^ (combination immunotherapy vs. sunitinib) and the Checkmate‐025 trial^17^ (second‐line nivolumab monotherapy vs. everolimus). However, this observation is limited by the small number of patients with RCC in our cohort. Additionally, both the Checkmate‐214 and ‐025 trials included patients with poor risk IMDC disease and thus our cohort, all intermediate risk by IMDC, likely had an improved prognosis at baseline. The disparity between the median PFS and OS for patients with RCC is noted within our population, a phenomenon also described in the Checkmate‐014 trial.^4^

Whilst ICIs have shown favourable activity in mesothelioma,^10^, ^33^ patients treated with pembrolizumab have a median OS rate of 18 months.^33^ The small number of patients with mesothelioma and the low 3‐ and 5‐year survival rates in our study precluded meaningful statistical analysis.

While our landmark OS rates are similar to published data, direct patient‐level comparison between our retrospective cohort and the published prospective clinical trial data could not be performed. The results of this study support the enduring benefits of immunotherapy which in some patients, extend to at least 5 years. The success of achieving durable responses and survival for patients also presents a potential workforce dilemma. With increasing survival, patients remain in oncology care for longer. This places higher demands on health services. This factor which has been considered in the context of rising chemotherapy^34^ uptake but not immunotherapy, which will likely increase the projected demands even further.

Our results support the findings of the CA209‐003 trial by Topalian et al. which similarly reported 5‐year OS rates among patients with melanoma, NSCLC and RCC treated with nivolumab.^35^ Topalian et al. also demonstrated that at 5 years, the presence of bone or liver metastases were associated with poor survival outcomes and ECOG status of 0 associated with improved survival outcomes. However, this study did not assess the correlation between irAE and OS and a larger proportion of their cohort were made up of patients with RCC and NSCLC.

The outcomes for different subgroups based on clinical characteristics in immunotherapy trials are variable. Further prospective data on prognostic and predictive patient factors are important to include in the development of further clinical trials. Other retrospective series have also demonstrated poor outcomes for patients with liver metastases treated with ICIs in populations of melanoma^36^, ^37^, ^38^ and NSCLC.^38^, ^39^ In our results, it is interesting to note that the presence of baseline liver and bone metastases were associated with reduced survival at 5 years but not associated with ORR or 5‐year PFS. It is postulated that ICIs have residual efficacy for a long duration and that these drugs could impact OS more than PFS even after the cessation of treatment.

Our data also confirm the previous observations of improved outcomes in patients who develop irAEs.^35^, ^40^ The clinical manifestation of irAEs may represent enhanced T‐cell activation correlating with an augmented overall immune response and improved anti‐tumour effect. Prior studies^41^, ^42^ have performed time‐dependant covariate analysis for irAE to reduce the influence of survivor bias. The retrospective nature of this cohort meant data was not available to perform this analysis. Nevertheless, both of these studies have demonstrated a relationship between irAE and PFS consistent with our results. Additionally, we note our cohort seemed to have a high rate of hypophysitis as an irAE. This is likely reflective of the retrospective nature and small numbers included in the cohort.

The results of our study did differ from some of the published literature regarding clinical predictors for immunotherapy response and long‐term survival. A history of smoking^43^, ^44^ has previously been associated with increased OS in patients who received immunotherapy for metastatic NSCLC. While our cohort did not demonstrate a benefit for smokers to receiving ICIs, the number of patients in our cohort who were current smokers was small. Regarding BMI, there is variable data regarding its impact on survival in patients receiving immunotherapy. Some studies have reported an association between higher BMI and improved survival^45^, ^46^ and a larger systematic review demonstrated no association.^47^ Whilst OS improves with immunotherapy for patients of both sexes, one published systematic review^48^ demonstrated a larger treatment effect from ICIs in males over females which was not demonstrated in our cohort. This highlights the need for prospective studies to include pre‐determined sub‐analyses in order to assess the prognostic values of these clinical factors.

To date, validated biomarkers for long term survival from immunotherapy have demonstrated variable reliability. Whilst PDL1 expression, tumour mutational burden (TMB) and Interferon‐gamma are thought to be predictive of response rates, these features have not been consistent across tumour types, demonstrate temporal and spatial heterogeneity^49^ and can change with multiple treatment lines. Recent assessment of TMB in more detail across multiple cancer types has demonstrated that this may not predictive of outcomes.^50^ Moreover, the current trend towards combining ICIs with chemotherapy, targeted agents and/or other novel agents may further confound the identification of useful predictive biomarkers. Our study and prior studies^35^ shed light on important predictive factors for survival outcomes with ICIs. Further studies with incorporation of clinical surrogates, tumour microenvironment assessment and multiomics data with specific focus on the organ‐specific site of disease are required for the development of predictive and dynamic biomarkers to ensure best utilization of ICIs and combination therapies moving forward.

### 4.1 Strengths and limitations

Our study correlates the enduring survival benefits of immunotherapy, reported in clinical trials, to a real‐world population with advanced cancer. This is supportive of prior studies of nivolumab.^35^ However, our cohort additionally verifies these findings in anti‐PD1, ipilimumab and combination immunotherapy. We also identify multiple clinical and anatomical factors of significant prognostic importance. These findings are not only directly informative, but are also hypothesis‐generating and expose areas for further research.

It is important to note that our study has several limitations. The retrospective nature of our study is associated with unavoidable selection bias. Although this was a multi‐centre study conducted at two large tertiary cancer centres, the sample size and heterogeneity of the cohort may influence the identification of predictive factors associated with immunotherapy and survival. Additionally, a majority of the study population was represented by patients with melanoma and NSCLC, reflective of the treatment paradigm within the study period. There were small numbers of patients with RCC and mesothelioma, and therefore some of the conclusions may be driven by the specific tumour phenotype rather than the clinical correlates.

## 5 CONCLUSIONS

Single agent anti‐PD1 therapy, anti‐CTLA4 therapy or combination immunotherapy is associated with long term survival in a real‐world population of both treatment naïve and pre‐treated patients with advanced melanoma, NSCLC and RCC. Identification of patients who will survive longer or develop an enduring response with immunotherapy in these solid organ tumours via additional markers of response is a clear unmet need. The results of our study suggest pre‐treatment ECOG and the development of irAEs may aid prognostic discussions, service planning and delivery for these patients.

## AUTHOR CONTRIBUTIONS

**Lauren Julia Brown:** Conceptualization (equal); data curation (lead); formal analysis (lead); investigation (equal); methodology (equal); project administration (equal); resources (equal); software (equal); validation (equal); writing – original draft (lead); writing – review and editing (equal). **Ines Pires Da Silva:** Formal analysis (supporting); methodology (supporting); supervision (equal); writing – original draft (supporting); writing – review and editing (equal). **Tania Moujaber:** Formal analysis (equal); methodology (equal); supervision (equal); writing – original draft (supporting); writing – review and editing (equal). **Bo Gao:** Conceptualization (equal); project administration (equal); supervision (equal); writing – review and editing (equal). **Rina hui:** Formal analysis (equal); methodology (equal); supervision (equal); writing – original draft (supporting); writing – review and editing (equal). **Howard Gurney:** Formal analysis (equal); project administration (equal); supervision (equal); writing – original draft (supporting); writing – review and editing (equal). **Matteo S. Carlino:** Conceptualization (equal); formal analysis (equal); project administration (equal); supervision (equal); writing – original draft (supporting); writing – review and editing (equal). **Adnan Nagrial:** Conceptualization (lead); data curation (equal); formal analysis (equal); investigation (equal); methodology (equal); project administration (lead); resources (equal); software (equal); supervision (lead); validation (equal); writing – original draft (equal); writing – review and editing (equal).

## FUNDING INFORMATION

No external funding was obtained for this specific project. LB is funded by the Westmead Hospital ICPMR Jerry Koutts Postgraduate Scholarship for 2022.

## CONFLICT OF INTEREST

There are no conflicts of interest. See below for the author disclosures: IDS: Consultant Advisor for MSD; Speaker honararia: Roche, Novartis, Bristol Myers Squibb. MSC: Consultant advisor: Amgen, Bristol‐Myers Squibb, Eisai, Ideaya, Merck Sharp and Dohme, Nektar, Novartis, Oncosec, Pierre‐Fabre, Qbiotics, Regeneron, Roche; honoraria: Bristol‐Myers Squibb, Merck Sharp and Dohme, Novartis. RH: Advisory board member for: AstraZeneca, Bristol‐Myers Squibb, Eisai, Eli Lilly, Merck, Merck Sharp and Dohme, Novartis, Oncosec, Pfizer, Roche, Seagen; Speaker honoraria from: AstraZeneca, Merck Sharp and Dohme, Novartis, Roche. TM: Advisory board for Merck/Pfizer. Speaker honoraria Amgen. BG: No relationships to disclose. HG: Advisory board member for: AstraZeneca, Bristol‐Myers Squibb, Eisai, Eli Lilly, Merck Serono, Merck Sharp and Dohme, Pfizer, Roche; Speaker honoraria from: AstraZeneca, Merck Sharp and Dohme, Pfizer. AN: Advisory board member for MSD, BMS, Roche, Astra Zeneca, PfizerMerck Serono.

## ETHICS STATEMENT

Ethics approval was obtained through the Western Sydney Local Health District Human Research and Ethics Committee.

## PATIENT CONSENT STATEMENT

Given the retrospective nature of this cohort, a waiver of consent was obtained by ethics approval.

## PERMISSION TO REPRODUCE MATERIAL FROM OTHER SOURCES

No material was reproduced from other sources in this manuscript.

## Supporting information


Figure S1.

Figure S2.
Figure S3.
Figure S4.
Figure S5.
Figure S6.
Figure S7.
Figure S8.
Figure S9.
Figure S10.
Figure S11.
Figure S12.
Figure S13.

Figure S14.
Figure S15.
Figure S16.
Figure S17.
Figure S18.

Table S1.
Table S2.
Table S3.
Table S4.
Table S5.
Table S6.
Table S7.

## Data Availability

The data that support the findings of this study are available on request from the corresponding author. The data are not publicly available due to privacy or ethical restrictions.
