# Supplementary figures and images for: Five‐year survival and clinical correlates among patients with advanced non‐small cell lung cancer, melanoma and renal cell carcinoma treated with immune check‐point inhibitors in Australian tertiary oncology centres
Source: Cancer Med. 2022 Nov 20;12(6):6788–801. doi: 10.1002/cam4.5468 (PMC10067054; doi:10.1002/cam4.5468)

Supplementary Figure 1

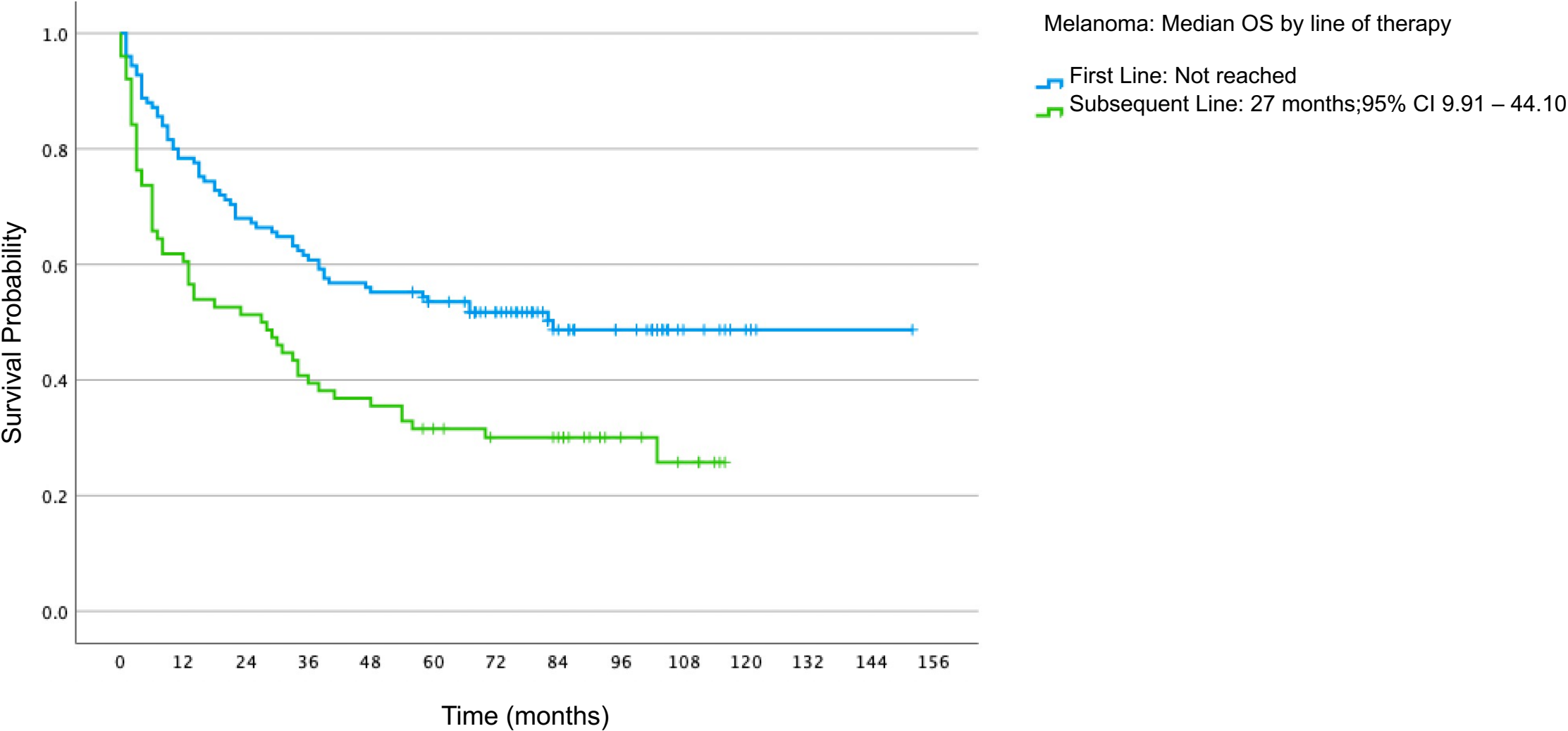

No. at risk

|                      |     |    |    |    |    |    |    |    |    |   |   |   |   |   |
|----------------------|-----|----|----|----|----|----|----|----|----|---|---|---|---|---|
| 1 <sup>st</sup> line | 125 | 98 | 85 | 77 | 70 | 62 | 49 | 30 | 22 | 9 | 4 | 1 | 1 | - |
| Subsequent line      | 77  | 47 | 39 | 31 | 28 | 23 | 19 | 18 | 9  | 5 | - | - | - | - |

Supplement: Supplementary file 1 — Figure S1. [file CAM4-12-6788-s001.pdf]
