# Supplementary material for: Five‐year survival and clinical correlates among patients with advanced non‐small cell lung cancer, melanoma and renal cell carcinoma treated with immune check‐point inhibitors in Australian tertiary oncology centres
Source: Cancer Med. 2022 Nov 20;12(6):6788–801. doi: 10.1002/cam4.5468 (PMC10067054; doi:10.1002/cam4.5468)
Supplement: Supplementary file 2 — Figure S2. Figure S3. Figure S4. Figure S5. Figure S6. Figure S7. Figure S8. Figure S9. Figure S10. Figure S11. Figure S12. Figure S13. [file CAM4-12-6788-s002.pdf]

Supplementary Figure 2

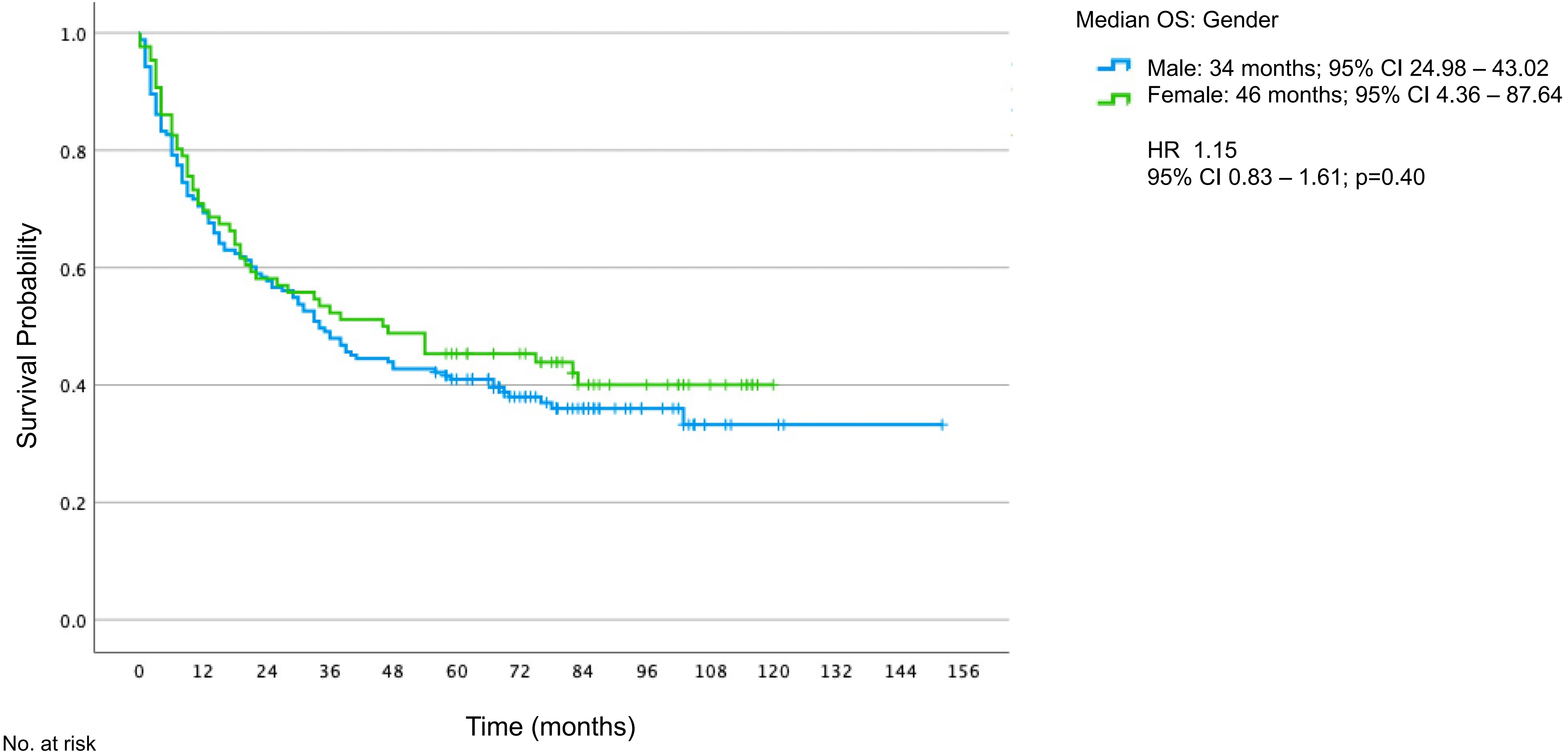

No. at risk

**Male**	173	122	101	85	76	66	44	29	16	5	3	1	1	-
**Female**	87	61	50	46	42	37	33	19	15	9	1	-	-	-

Supplementary Figure 3

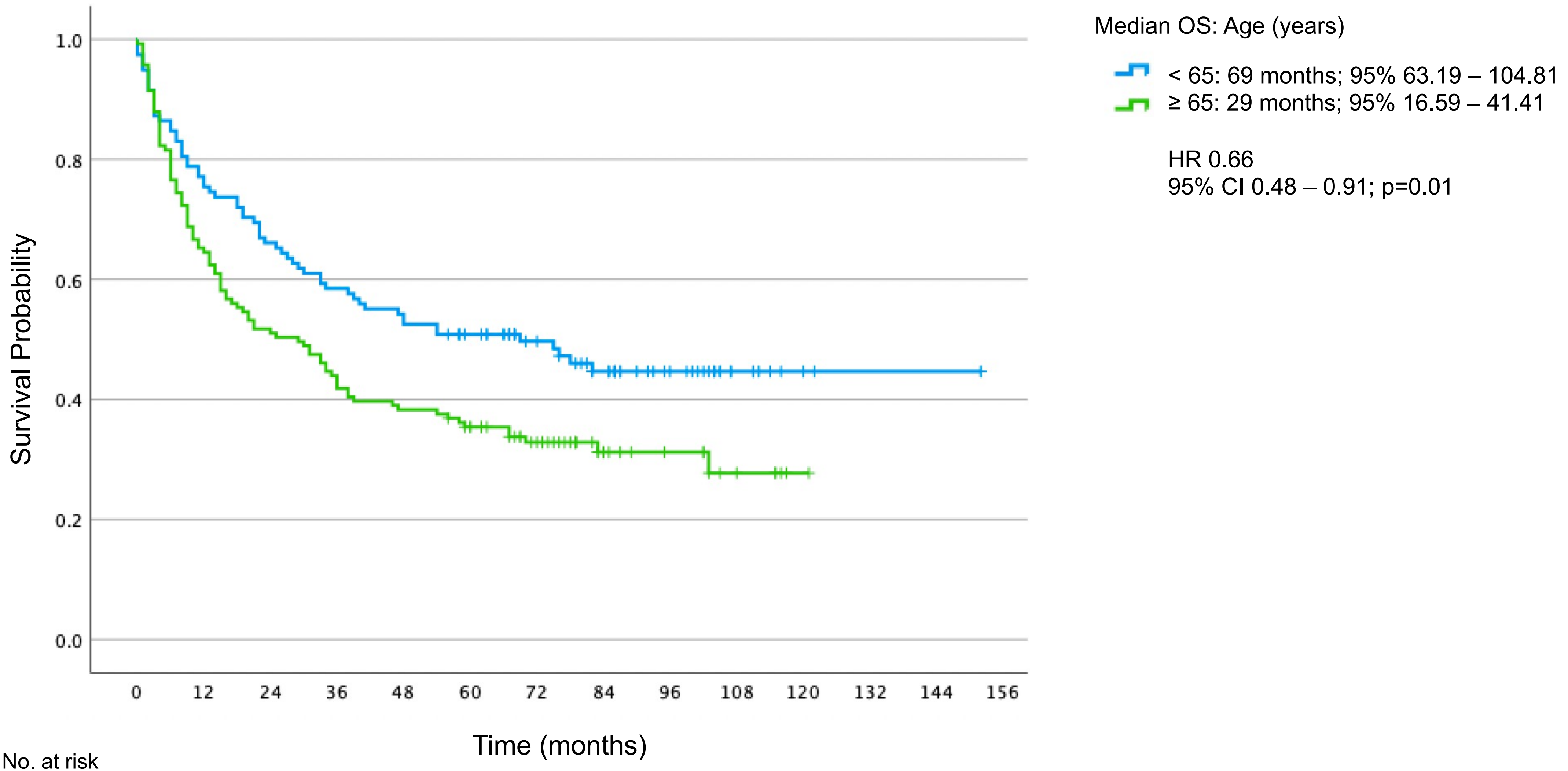

No. at risk

< 65	119	90	78	69	64	55	42	31	20	8	3	1	1	-
≥ 65	141	92	73	62	54	48	35	17	11	6	1	-	-	-

Supplementary Figure 4

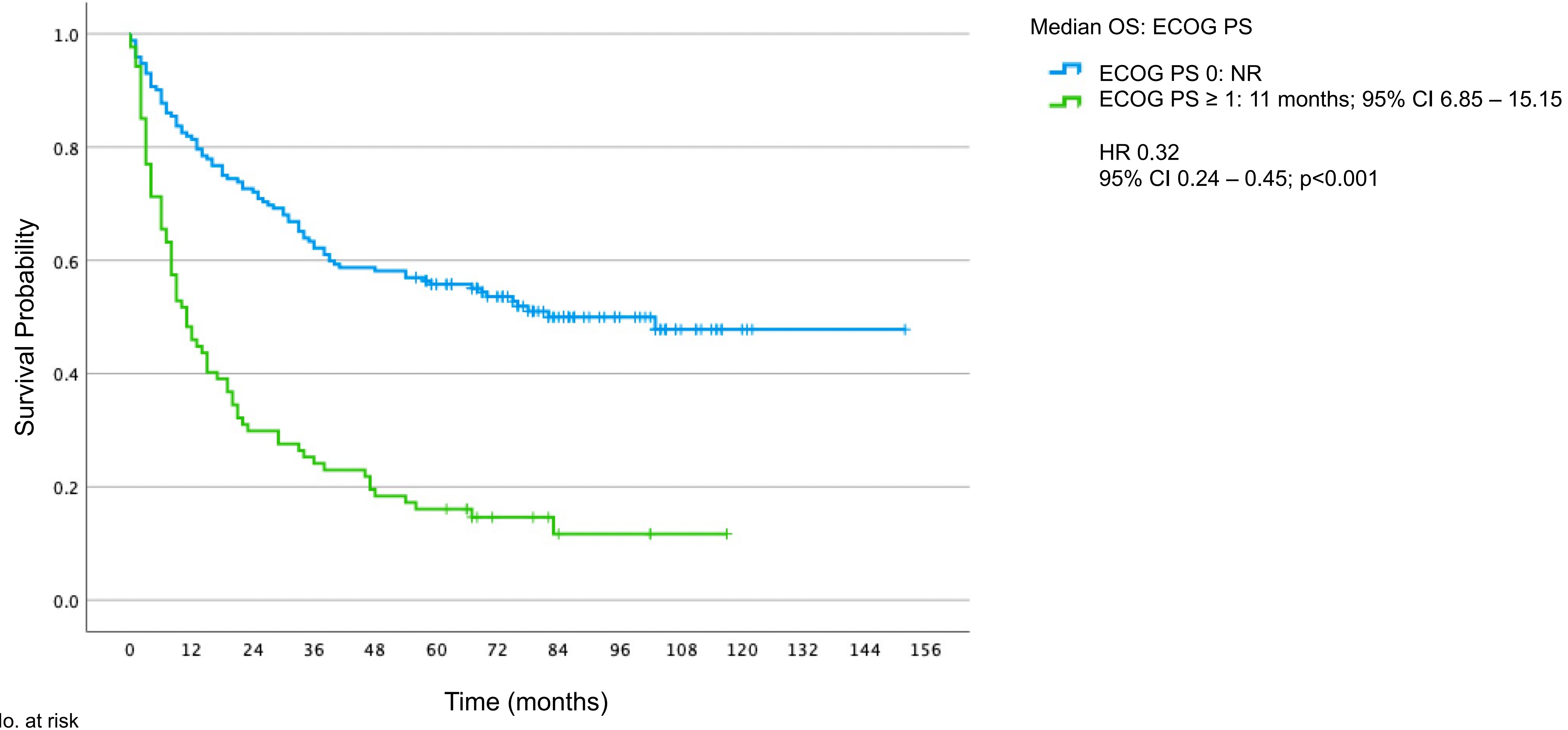

No. at risk

**ECOG 0**	173	140	125	109	89	70	44	28	13	4	1	1	1	-
**ECOG ≥1**	87	42	26	22	17	14	7	4	3	1	-	-	-	-

Supplementary Figure 5

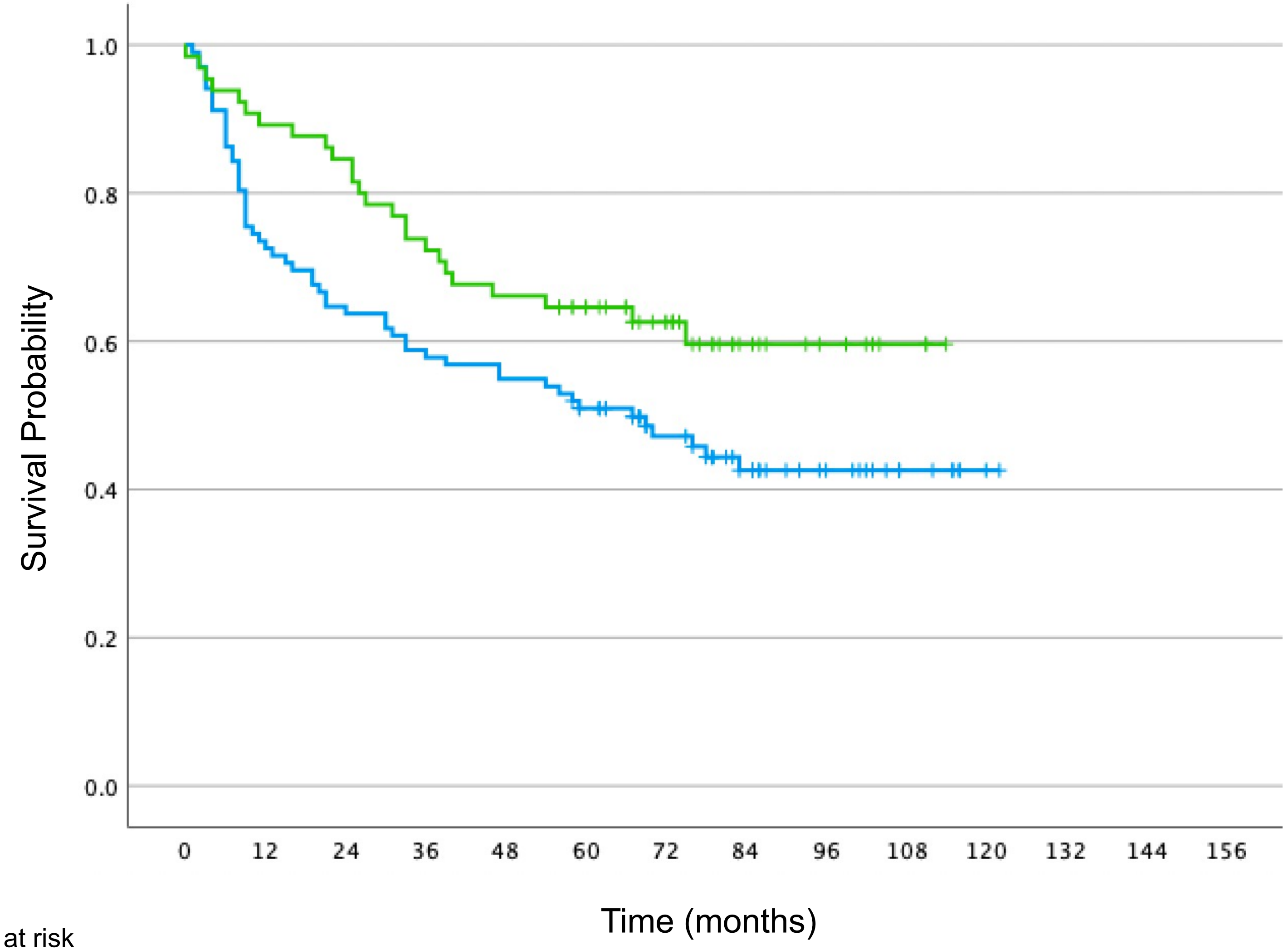

Median OS: BMI

Normal / Underweight: 67 months; 95% 36.56 – 97.44
Obese / Overweight: NR

HR 1.64
95% CI 1.02 – 2.63; p=0.04

No. at risk														
**BMI Normal / Underweight**	102	75	66	60	56	49	35	23	15	7	2	-	-	-
**BMI Obese / Overweight**	65	58	55	48	43	38	27	12	7	3	-	-	-	-

Supplementary Figure 6

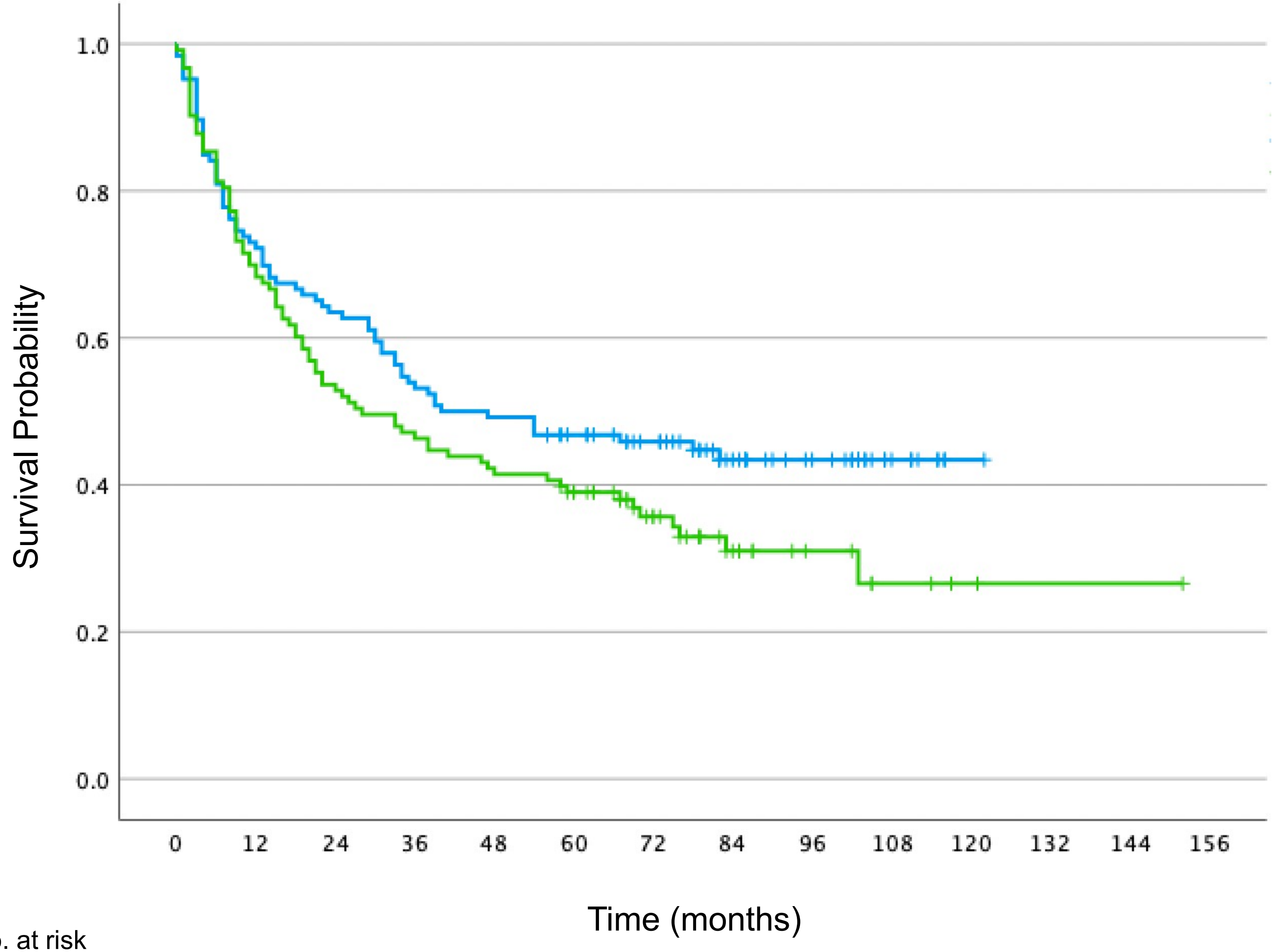

Median OS: Smoking Status

Never Smoker: 40 months; 95% CI 6.15 – 73.85
Ex-smoker / Current Smoker: 28 months; 95% CI 13.79 – 42.21

HR 0.76
95% CI 0.55 – 1.05; p=0.09

No. at risk														
**Smoking -**	126	92	80	69	62	54	45	30	21	9	1	-	-	-
**Smoking +**	124	85	66	58	52	46	29	15	8	4	2	1	1	-

Supplementary Figure 7

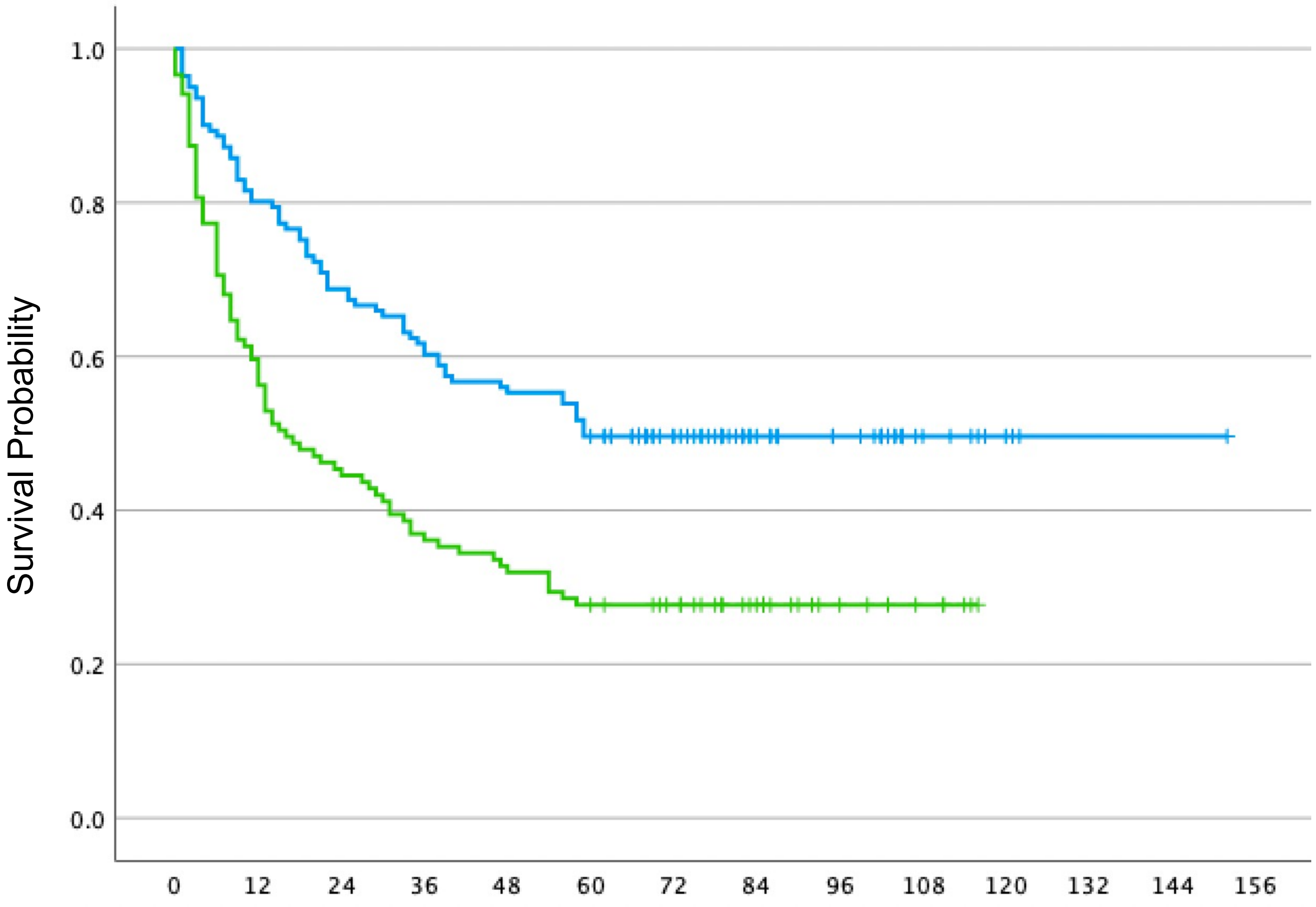

Time (months)

Median OS: Prior Therapies

0: No Prior therapy: 59 months
≥1 Prior therapies: 16 months; 95% CI 6.84 – 25.16

HR 0.84
95% CI 0.49 – 1.46; p=0.54

No. at risk

0	141	113	97	87	79	70	49	30	22	9	4	1	1	-
≥1	119	71	54	44	39	33	28	18	9	5	-	-	-	-

Supplementary Figure 8

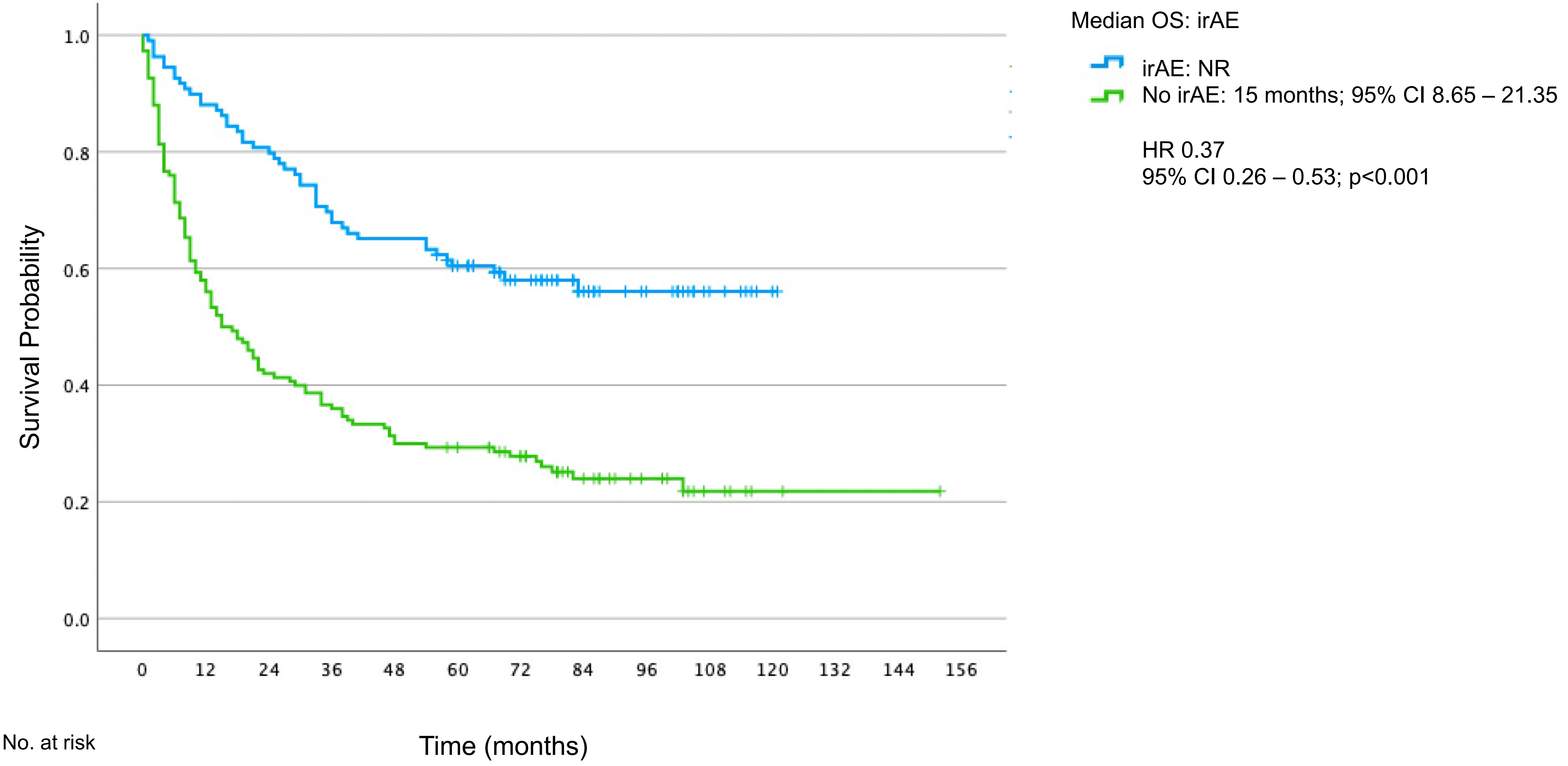

No. at risk														
irAE	109	96	88	76	71	60	41	27	18	8	2	-	-	-
No irAE	151	86	63	55	47	43	36	21	13	6	2	1	1	-

Supplementary Figure 9

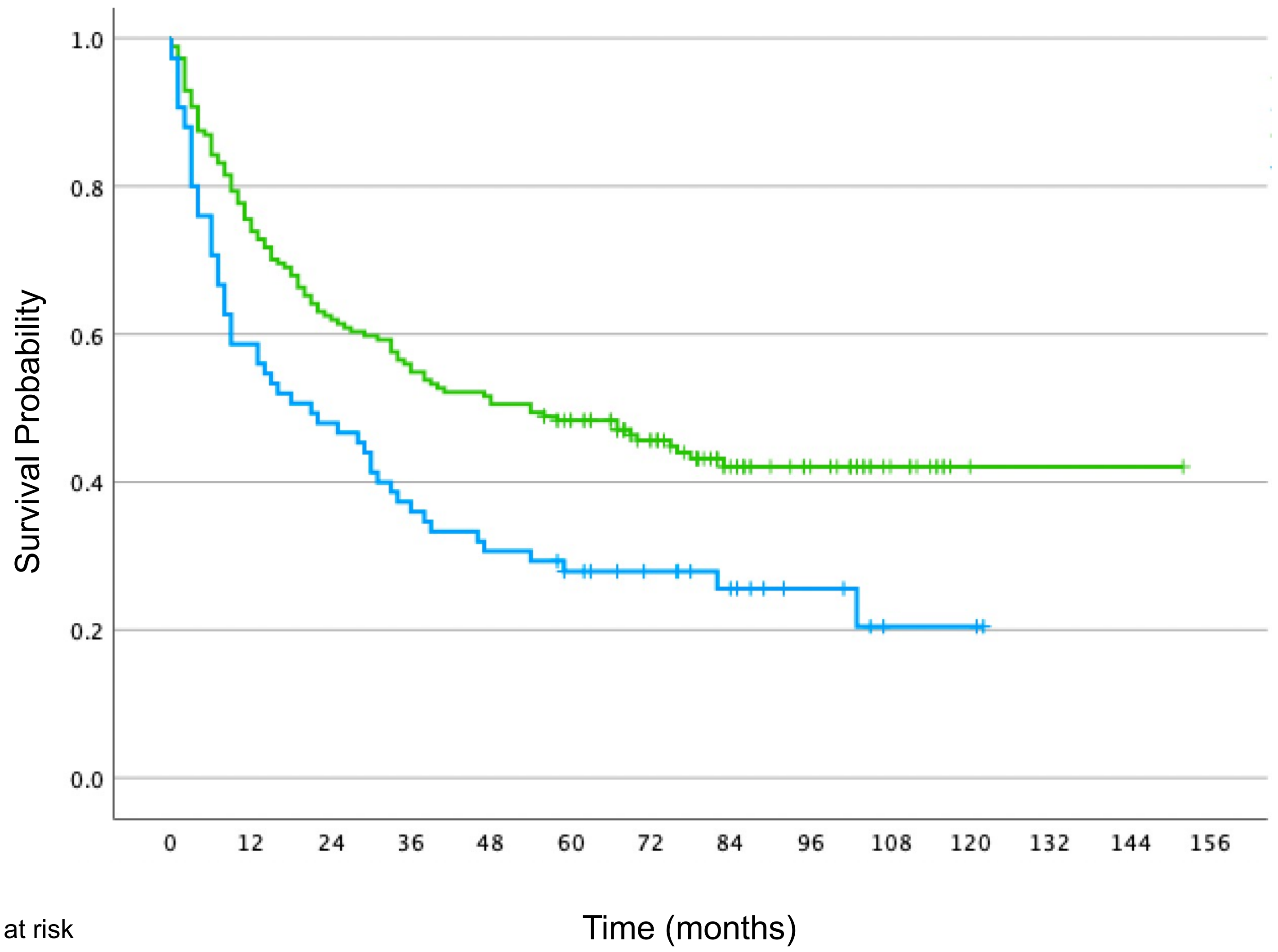

Median OS: Liver metastases

Liver Metastases: 21 months; 95% CI 5.91 – 36.09
No Liver Metastases: 54 months; 95% CI 28.05 – 79.95

HR 1.71
95% CI 1.23 – 2.36; p<0.001

No. at risk														
**Liver mets +**	75	44	36	28	19	15	11	6	2	2	-	-	-	-
**Liver mets -**	185	139	114	103	95	84	62	37	25	12	2	1	1	-

Supplementary Figure 10

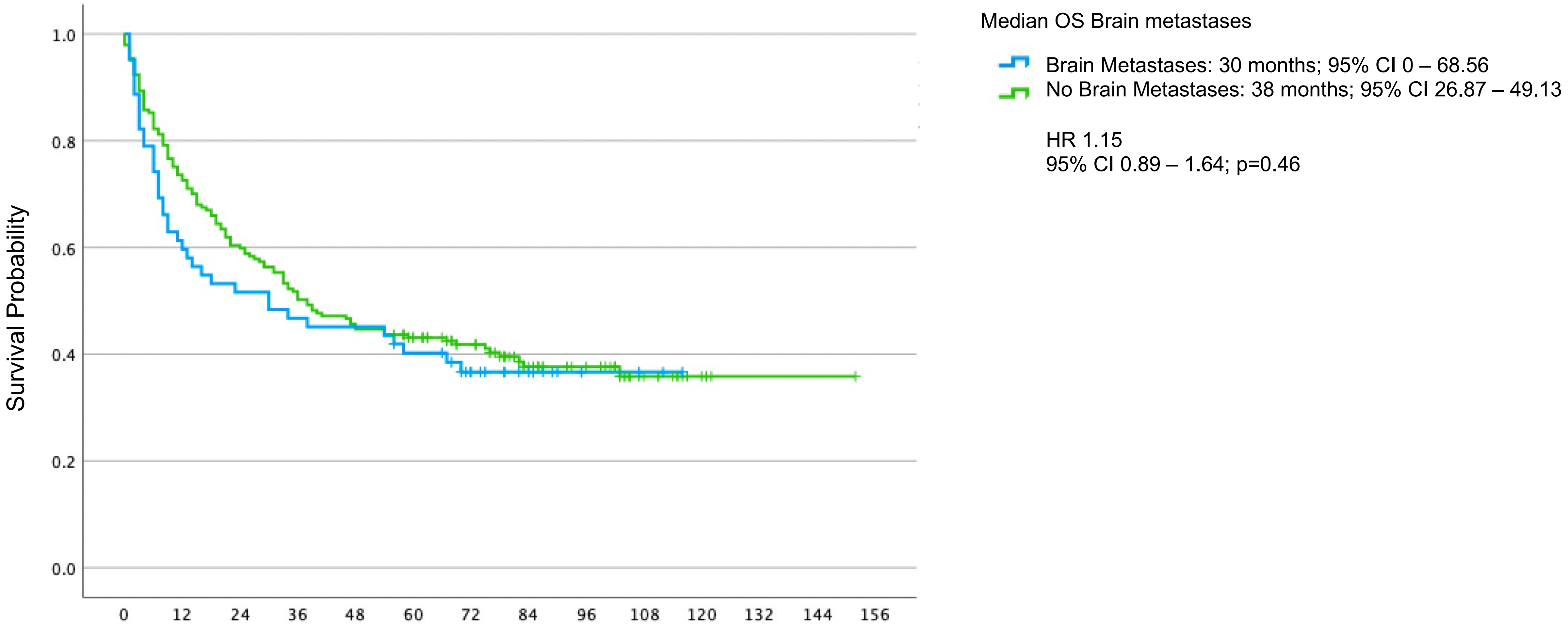

Time (months)

No. at risk														
**Brain mets +**	62	38	32	29	28	18	11	3	2	-	-	-	-	-
**Brain mets -**	198	144	119	102	90	79	59	37	28	12	4	1	1	-

Supplementary Figure 11

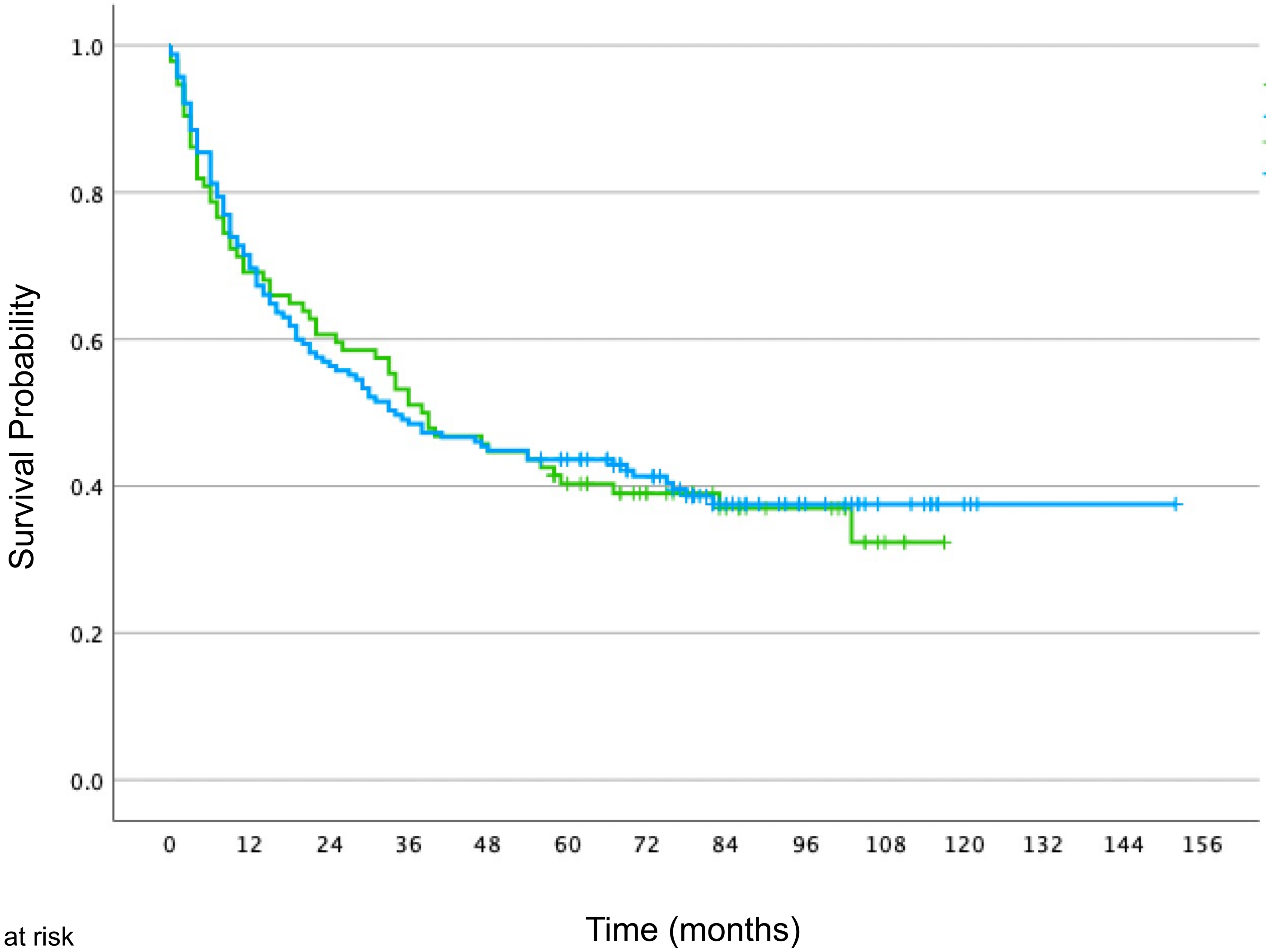

Median OS Lung metastases

Lung Metastases: 34 months; 95% CI 18.45 – 49.55
No Lung Metastases: 38 months; 95% CI 23.75 – 52.25

HR 1.00
95% CI 0.72 – 1.37; p=0.98

No. at risk

**Lung mets +**	166	117	94	81	75	68	51	31	19	10	4	1	1	-
**Lung mets -**	94	65	57	50	43	35	26	17	12	4	-	-	-	-

Supplementary Figure 12

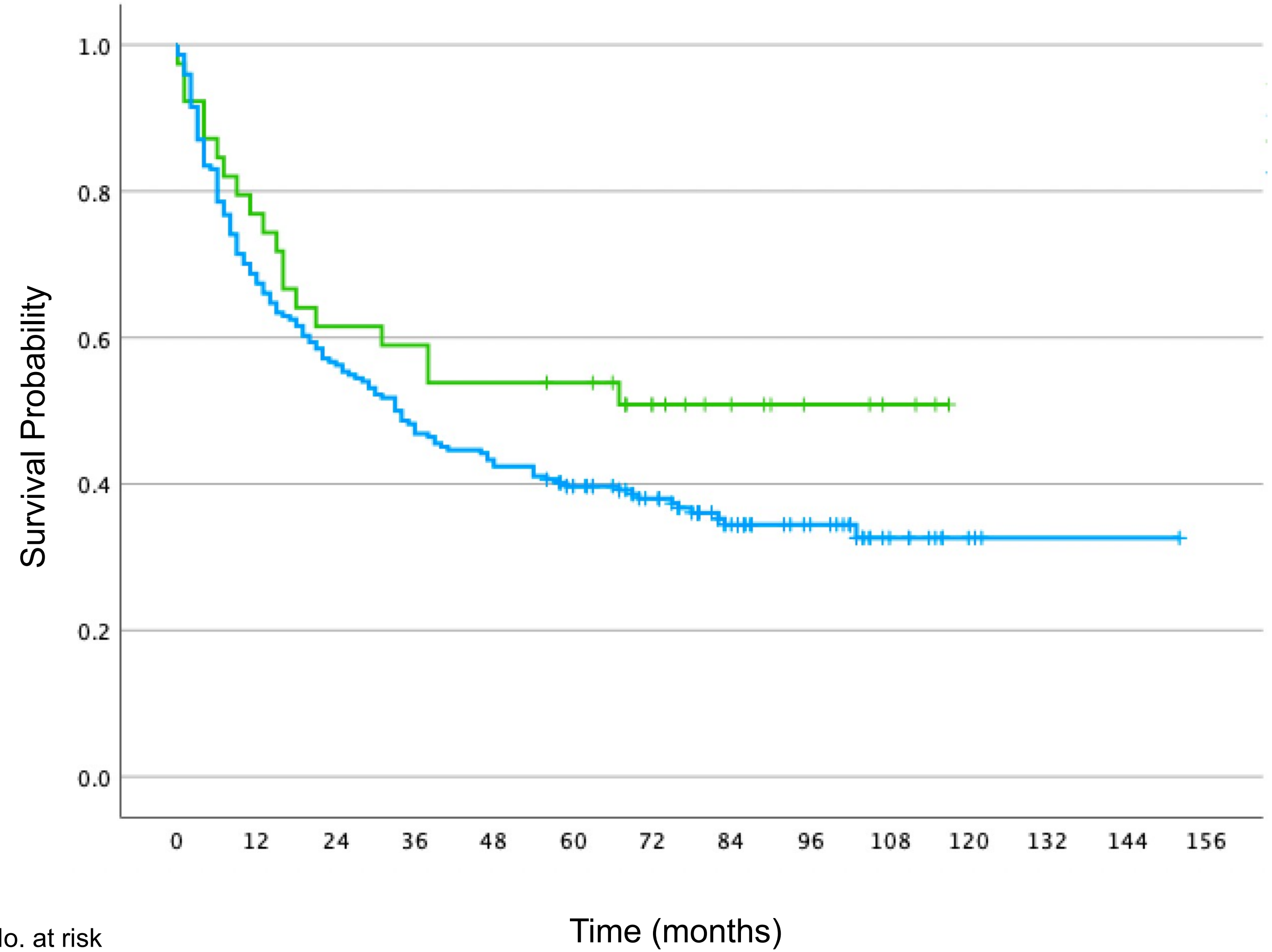

Median OS: Lymph Node metastases

LN Metastases: 33 months; 95% CI 23.22 – 42.78
No LN Metastases: NR

HR 1.66
95% CI 0.99 – 2.78; p=0.05

No. at risk														
**LN mets +**	224	153	127	108	97	83	63	39	26	11	4	1	1	-
**LN mets -**	36	29	24	23	21	20	14	9	5	3	-	-	-	-

Supplementary Figure 13

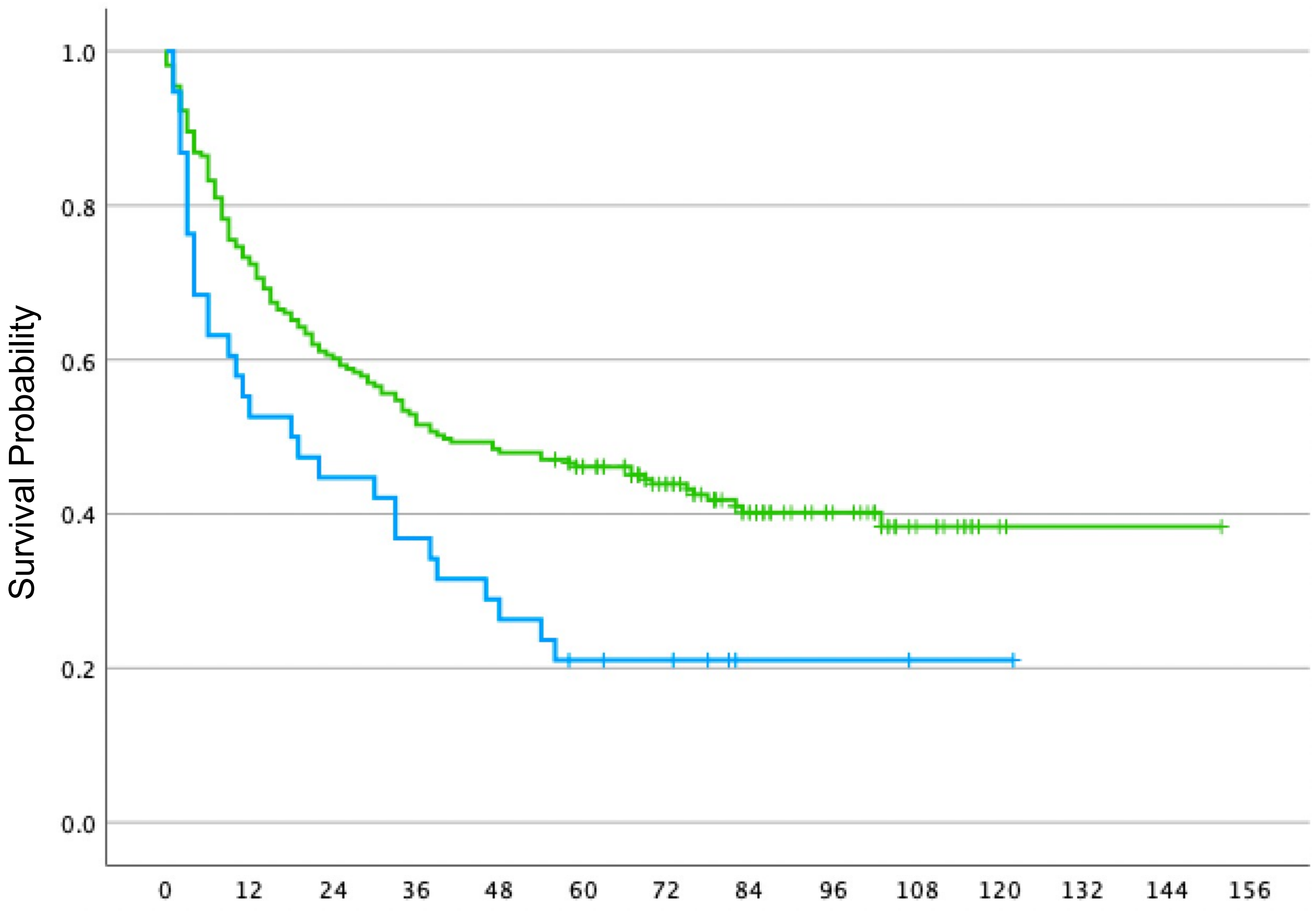

Median OS: Bone metastases

Bone Metastases: 18 months; 95% CI 1.39 -34.61
No Bone Metastases: 40 months; 95% CI 17.58 – 62.42

HR 1.76
95% CI 1.18 – 2.63; p<0.01

No. at risk														
**Bone mets +**	39	20	17	14	11	7	6	2	2	1	1	-	-	-
**Bone mets -**	221	162	134	117	107	96	71	46	29	13	3	1	1	-
