# Supplementary material for: Five‐year survival and clinical correlates among patients with advanced non‐small cell lung cancer, melanoma and renal cell carcinoma treated with immune check‐point inhibitors in Australian tertiary oncology centres
Source: Cancer Med. 2022 Nov 20;12(6):6788–801. doi: 10.1002/cam4.5468 (PMC10067054; doi:10.1002/cam4.5468)
Supplement: Supplementary file 3 — Figure S14. Figure S15. Figure S16. Figure S17. Figure S18. [file CAM4-12-6788-s003.pdf]

Supplementary Figure 14

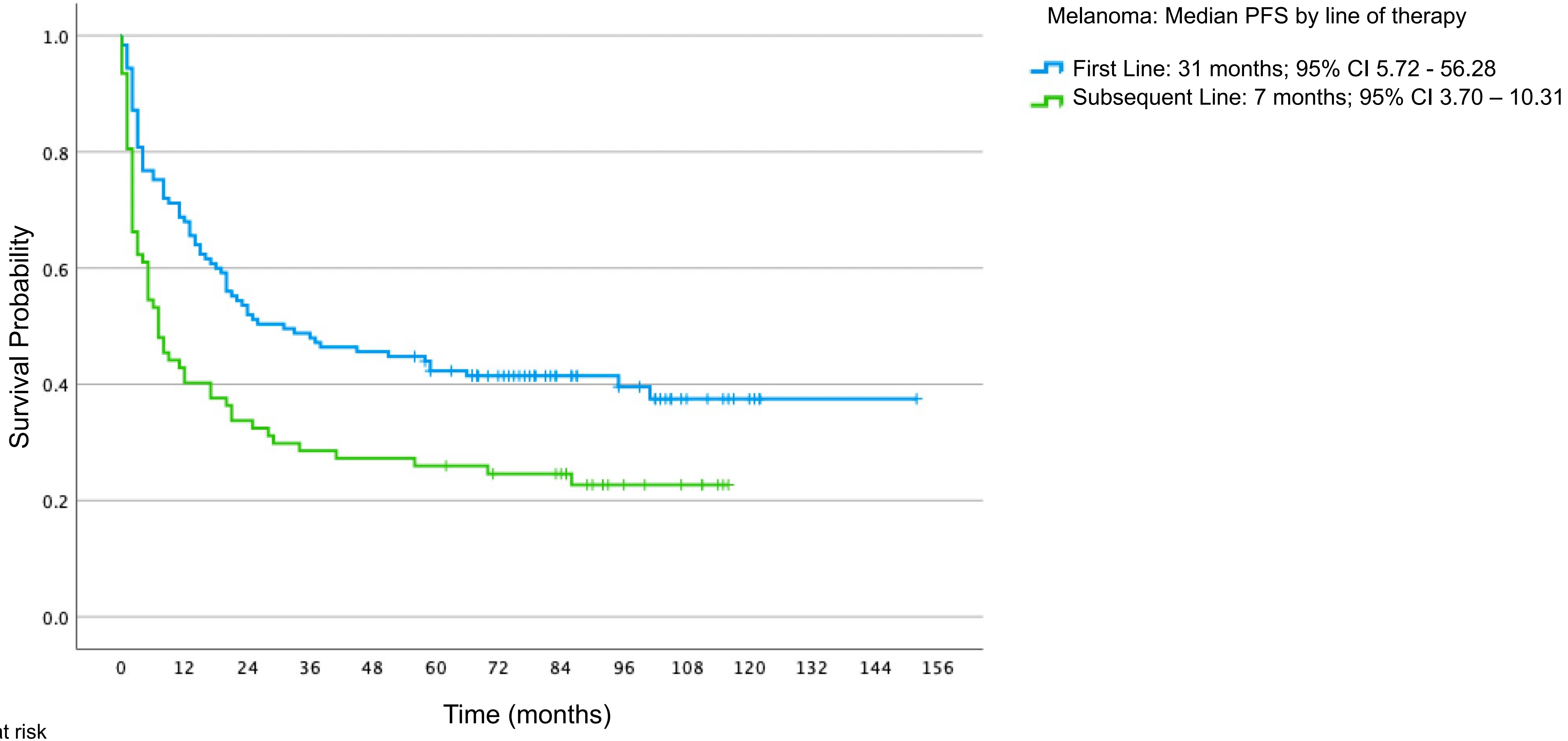

No. at risk

**1st line**	125	86	67	61	57	50	40	26	20	10	5	1	1	-
**Subsequent line**	77	33	26	22	21	20	17	16	8	5	-	-	-	-

Supplementary Figure 15

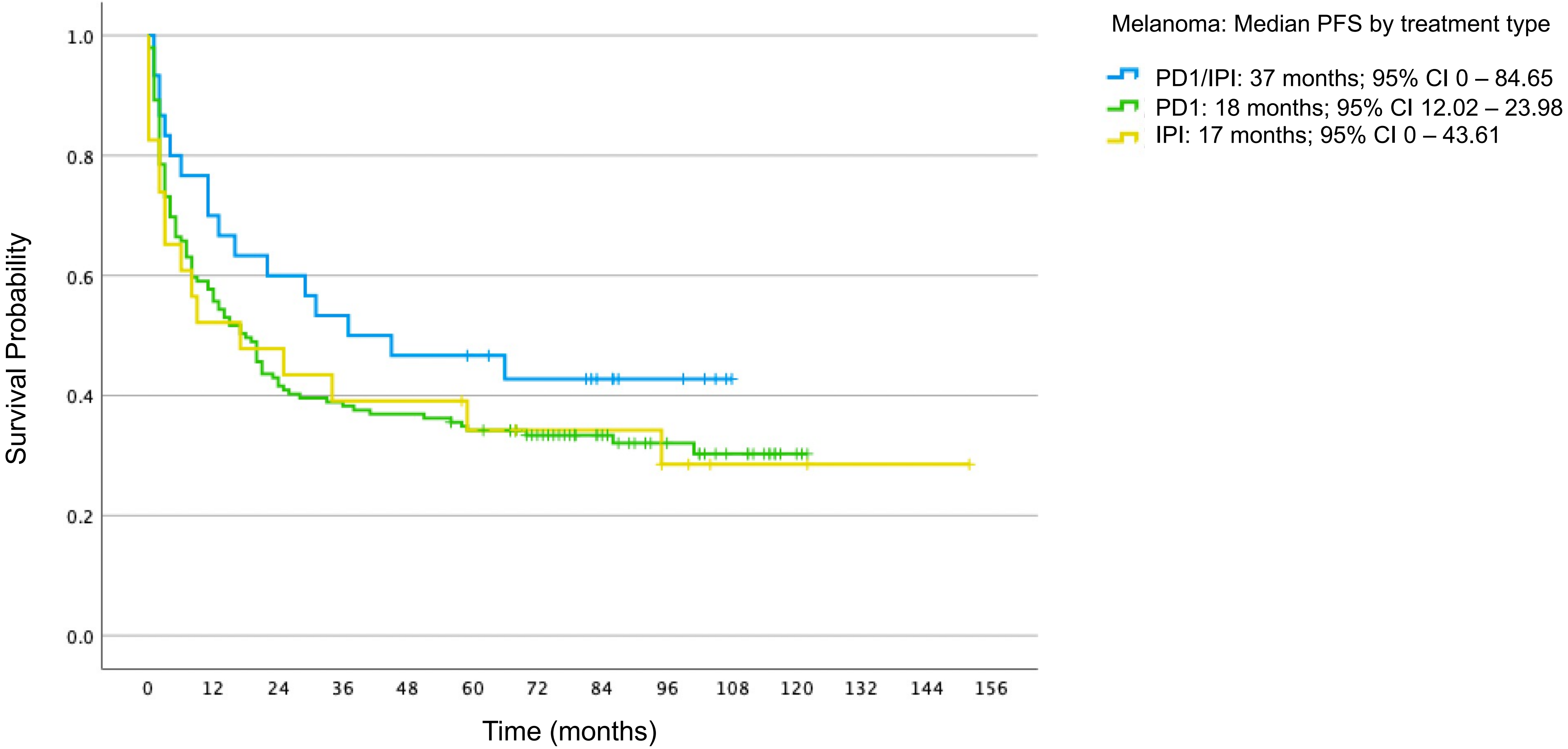

No. at risk

IPI/PD1	30	21	18	16	14	13	11	8	5	1	-	-	-	-
PD1	149	86	64	58	55	50	40	28	19	12	3	-	-	-
IPI	23	12	11	9	9	7	6	6	4	2	2	1	1	-

Supplementary Figure 16

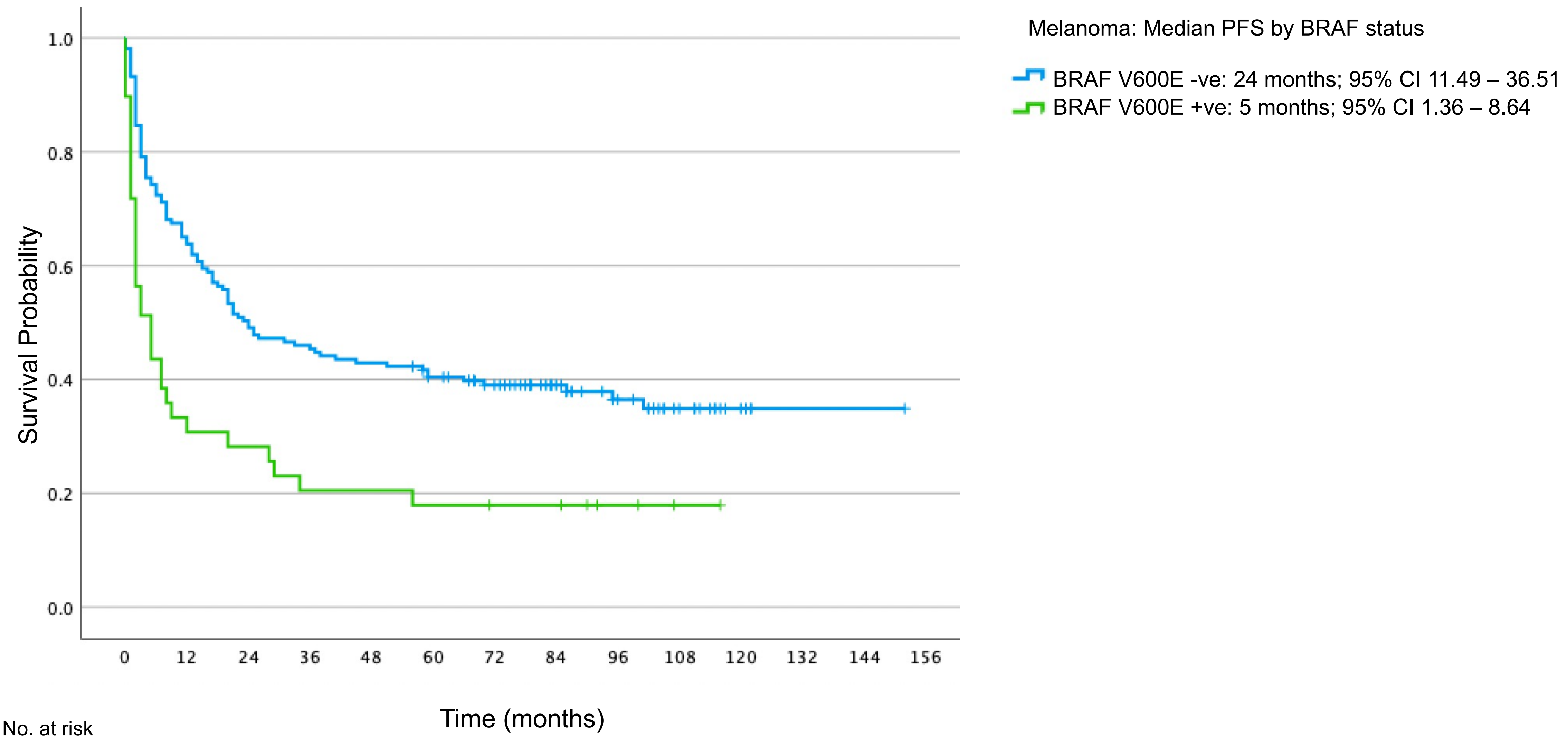

No. at risk														
**BRAF -**	163	106	82	75	70	63	51	36	25	14	5	1	1	-
**BRAF +**	39	13	11	8	8	7	6	6	3	1	-	-	-	-

Supplementary Figure 17

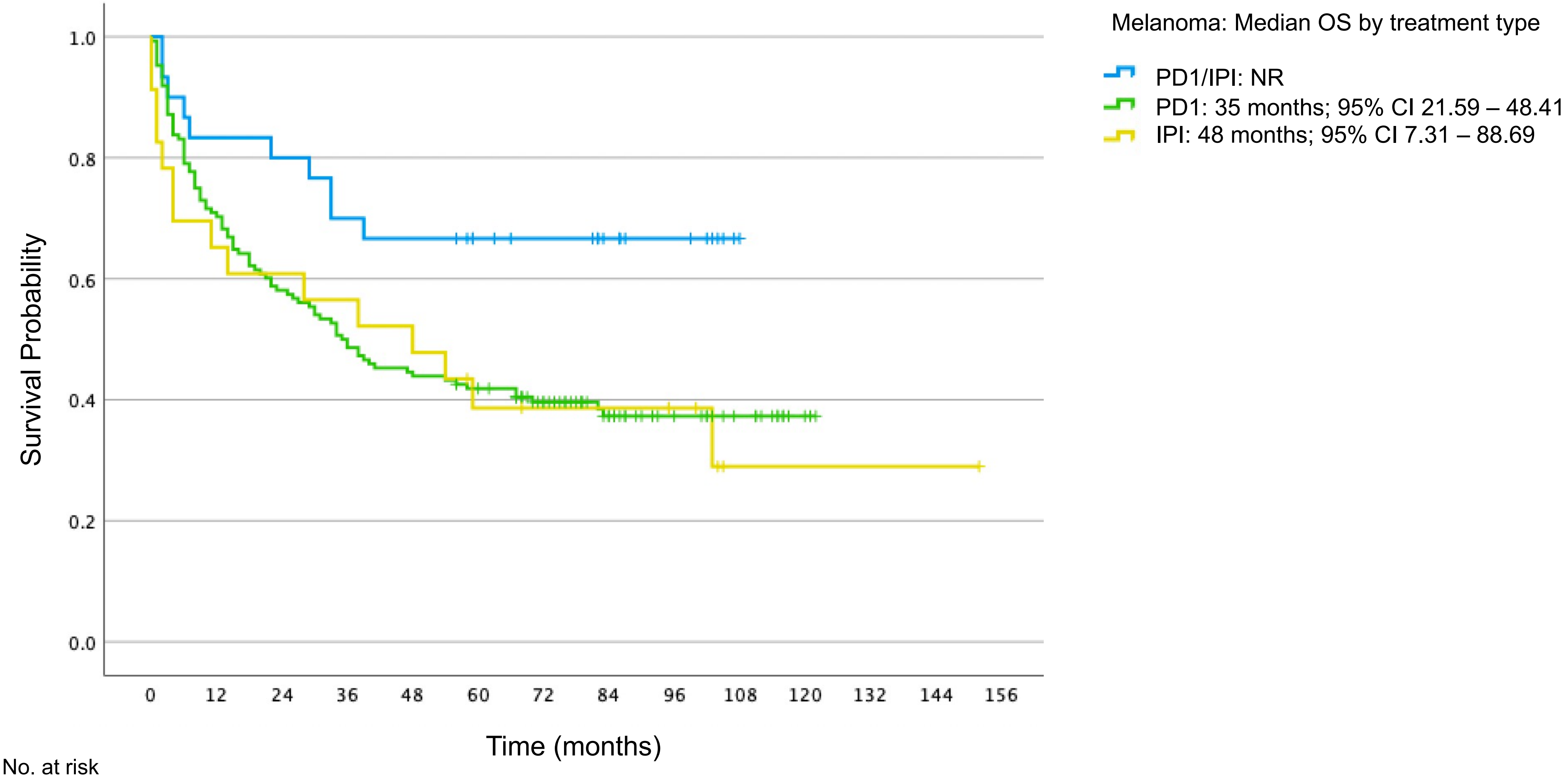

No. at risk														
IPI/PD1	30	25	24	21	20	16	14	10	7	1	-	-	-	-
PD1	149	105	86	74	66	61	47	31	19	12	3	-	-	-
IPI	23	15	14	13	12	8	7	7	5	1	-	-	-	-

Supplementary Figure 18

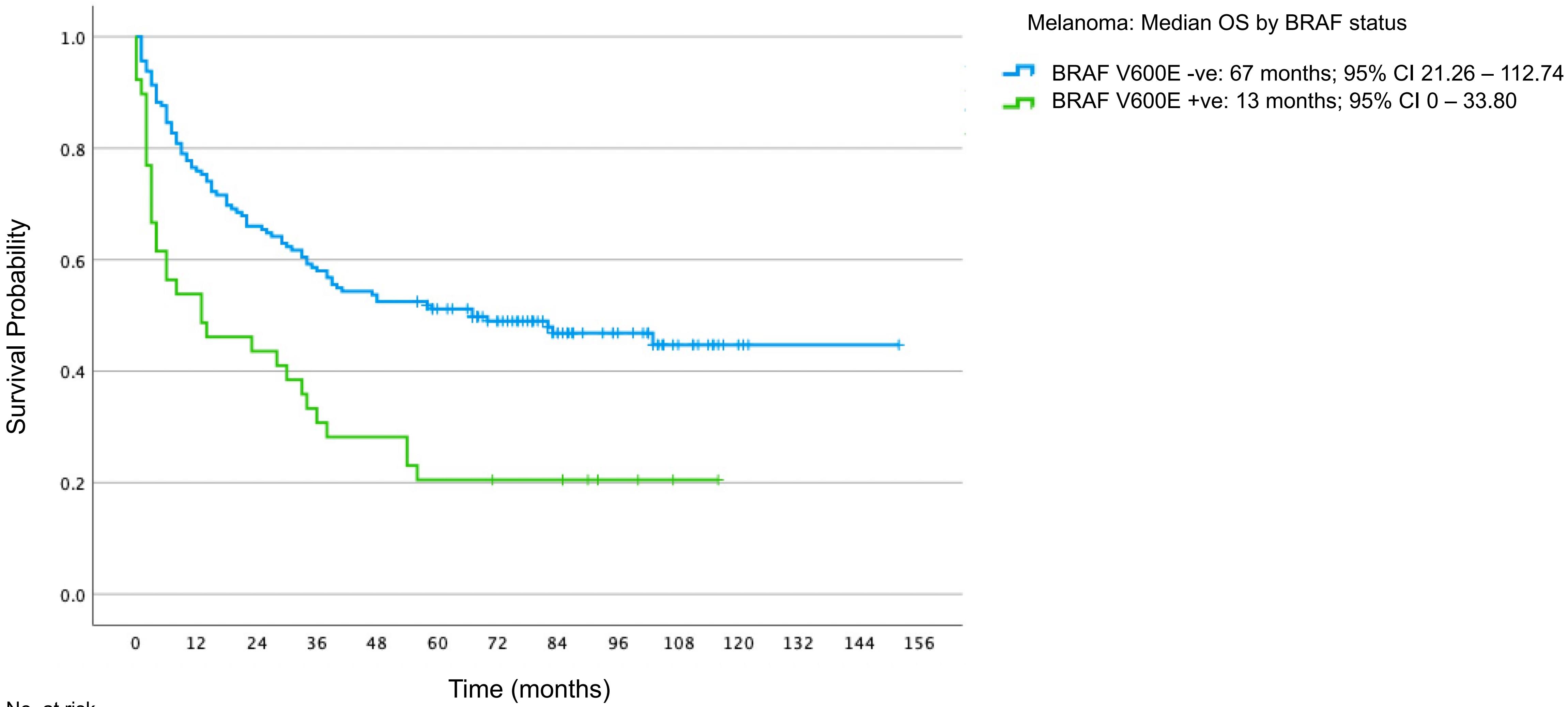

No. at risk

BRAF -	163	124	107	95	87	77	61	41	28	13	4	1	1	-
BRAF +	39	21	17	13	11	8	7	7	3	1	-	-	-	-
