# Supplementary material for: Five‐year survival and clinical correlates among patients with advanced non‐small cell lung cancer, melanoma and renal cell carcinoma treated with immune check‐point inhibitors in Australian tertiary oncology centres
Source: Cancer Med. 2022 Nov 20;12(6):6788–801. doi: 10.1002/cam4.5468 (PMC10067054; doi:10.1002/cam4.5468)
Supplement: Supplementary file 4 — Table S1. Table S2. Table S3. Table S4. Table S5. Table S6. Table S7. [file CAM4-12-6788-s004.docx]

**Supplementary Tables**

**Supplementary Table 1: Types of Immunotherapy by Tumour type**

|  | Pembrolizumab 10mg/kg 3 weekly OR 200mg 3 weekly (n) | Nivolumab 3mg/kg 2 weekly OR 240mg 2 weekly (n) | Ipilimumab 3mg/kg 3 weekly (n) | Pembrolizumab 2m/kg + Ipilimumab 1mg/kg (n) | Nivolumab 3mg/kg + Ipilimumab 1mg/kg (n) | Nivolumab 1mg/kg + Ipilimumab 3mg/kg (n) | Nivolumab 360mg 3 weekly + Ipilimumab 1mg/kg 6 weekly (n) |
| --- | --- | --- | --- | --- | --- | --- | --- |
| Melanoma | 144 | 5 | 23 | 18 | 0 | 12 | 0 |
| NSCLC | 1 | 40 | 0 | 0 | 0 | 2 | 3 |
| RCC | 0 | 3 | 0 | 0 | 9 | 0 | 0 |
| Mesothelioma | 4 | 0 | 0 | 0 | 0 | 0 | 0 |
| Total Patients | 149 | 48 | 23 | 18 | 9 | 14 | 3 |

n: Number; NSCLC: Non-small cell lung cancer; RCC: Renal cell carcinoma

**Supplementary Table 2: Univariate and multivariate analysis with Hazard Ratio for Overall Response Rate**

|  |  |  | Univariate Analysis | | Multivariate Analysis | |
| --- | --- | --- | --- | --- | --- | --- |
| Variable | **Patients**  **(n=260**^†^) | **ORR, %** | **Hazard Ratio (95% CI)** | **P value** | **Hazard Ratio (95% CI)** | **P value** |
| Age, y  < 65  ≥ 65 | 119  141 | 60.5  44.0 | 0.65 (0.45 – 0.93) | 0.04 | 0.63 (0.35 -1.13) | 0.12 |
| Sex  Male  Female | 173  87 | 51.4  51.7 | 1.08 (0.74 – 1.56) | 0.70 | 0.90 (0.53 – 1.54) | 0.71 |
| ECOG PS  0  ≥1 | 173  87 | 63.6  27.6 | 0.34 (0.24 – 0.48) | <0.001 | 0.41 (0.23 – 0.73) | <0.01 |
| BMI  Normal/underweight  Overweight | 102^‡^  65^‡^ | 59.8  67.7 | 0.74 (0.44 -1.26) | 0.27 | 1.07 (0.61 – 1.88) | 0.81 |
| Smoking status  Never smoker  Current/prior smoker | 126^‡^  124^‡^ | 57.1  46.0 | 0.73 (0.51 – 1.04) | 0.12 | 0.58 (0.33 -1.04) | 0.07 |
| irAE  Yes  No | 109  151 | 73.4  35.8 | 0.30 (0.20 – 0.45) | <0.001 | 0.37 (0.20 – 0.67) | <0.001 |
| Prior systemic therapies  0  ≥1 | 141  119 | 64.5  36.1 | 0.41 (0.29 – 0.59) | <0.001 | 0.80(0.42 – 1.52) | 0.49 |
| Bone metastases  Yes  No | 39  221 | 41.0  53.4 | 1.52 (0.97 – 2.40) | 0.07 | 1.43 (0.74 – 2.77) | 0.29 |
| Liver metastases  Yes  No | 75  185 | 42.7  55.1 | 1.53 (1.05 – 2.21) | 0.03 | 1.26 (0.70 – 2.30) | 0.44 |
| Lung metastases  Yes  No | 166  94 | 52.4  50.0 | 0.97 (0.67 – 1.39) | 0.86 | 1.10 (0.59 – 2.02) | 0.77 |
| LN metastases  Yes  No | 224  36 | 49.1  66.7 | 1.78 (0.98 – 3.22) | 0.06 | 2.57 (0.86 – 7.68) | 0.09 |
| Brain metastases  Yes  No | 62  198 | 41.9  54.5 | 1.43 (0.97 – 2.11) | 0.07 | 1.83 (1.02 – 3.29) | 0.04 |

n: Number; ORR: Overall response rate; HR: Hazard ratio; CI: Confidence interval; ECOG PS: European Cooperative Oncology Group Performance Status; BMI: Body Mass Index; irAE: Immune-related adverse event; LN: Lymph nodes

^†^ Excludes patients with mesothelioma (n=4)

^‡^ Data missing for some patients

**Supplementary Table 3: Univariate and multivariate analysis with Hazard Ratio for 5-year Progression Free Survival**

|  |  |  | Univariate Analysis | | Multivariate Analysis | |
| --- | --- | --- | --- | --- | --- | --- |
| Variable | **Patients**  **(n=260**^†^) | **5-year PFS, %** | **Hazard Ratio (95% CI)** | **P value** | **Hazard Ratio (95% CI)** | **P value** |
| Age, y  < 65  ≥ 65 | 119  141 | 37.0  24.8 | 0.74 (0.55 – 1.00) | 0.05 | 0.81 (0.52 – 1.26) | 0.35 |
| Sex  Male  Female | 173  87 | 28.9  33.3 | 1.14 (0.83 – 1.56) | 0.42 | 1.08 (0.70 – 1.66) | 0.74 |
| ECOG PS  0  ≥1 | 173  87 | 39.3  12.6 | 0.42 (0.31 – 0.56) | <0.001 | 0.53 ( 0.33 – 0.84) | <0.01 |
| BMI  Normal/underweight  Overweight | 102^‡^  65^‡^ | 41.2  38.5 | 1.06 (0.71 – 1.58) | 0.78 | 0.92 (0.60 – 1.40) | 0.92 |
| Smoking status  Never smoker  Current/prior smoker | 126^‡^  124^‡^ | 34.1  27.4 | 0.82 (0.61 – 1.11) | 0.20 | 0.75 (0.49 – 1.15) | 0.19 |
| irAE  Yes  No | 109  151 | 43.1  21.2 | 0.46 (0.34 – 0.63) | <0.001 | 0.66 (0.42 – 1.02) | 0.06 |
| Prior systemic therapies  0  ≥1 | 141  119 | 38.3  21.0 | 0.53 (0.40 – 0.72) | <0.001 | 0.79 (0.49 – 1.28) | 0.79 |
| Bone metastases  Yes  No | 39  221 | 12.8  33.5 | 1.66 (1.14 – 2.40) | <0.01 | 1.59 (0.96 – 2.64) | 0.07 |
| Liver metastases  Yes  No | 75  185 | 18.7  35.1 | 1.57 (1.15 – 2.14) | <0.01 | 1.46 (0.93 – 2.29) | 0.10 |
| Lung metastases  Yes  No | 166  94 | 31.3  28.7 | 0.91 (0.73 – 1.33) | 0.91 | 1.08 (0.69 – 1.68) | 0.74 |
| LN metastases  Yes  No | 224  36 | 28.1  44.4 | 1.62 (1.01 – 2.57) | 0.04 | 1.69 (0.84 – 3.45) | 0.15 |
| Brain metastases  Yes  No | 62  198 | 29.0  30.8 | 1.15 (0.82 0 1.61) | 0.44 | 1.31 (0.49 – 1.28) | 0.28 |

n: Number; PFS: Progression free survival; HR: Hazard ratio; CI: Confidence interval; ECOG PS: European Cooperative Oncology Group Performance Status; BMI: Body Mass Index; irAE: Immune-related adverse event; LN: Lymph nodes

^†^ Excludes patients with mesothelioma (n=4)

^‡^ Data missing for some patients

**Supplementary Table 4: Univariate and multivariate analysis with Hazard Ratio for 3-year Overall Survival**

|  |  |  | Univariate Analysis | | Multivariate Analysis | |
| --- | --- | --- | --- | --- | --- | --- |
| Variable | **Patients**  **(n=260**^†^) | **3-year OS, %** | **Hazard Ratio (95% CI)** | **P value** | **Hazard Ratio (95% CI)** | **P value** |
| Age, y  < 65  ≥ 65 | 119  141 | 58.3  43.3 | 0.65 (0.45 – 0.92) | 0.02 | 0.83 (0.46 – 1.49) | 0.53 |
| Sex  Male  Female | 173  87 | 49.1  52.9 | 1.13 (0.78 – 1.63) | 0.53 | 1.11 (0.63 – 1.95) | 0.73 |
| ECOG PS  0  ≥1 | 173  87 | 63.0  25.3 | 0.33 (0.23 – 0.47) | <0.001 | 0.38 (0.21 – 0.71) | <0.01 |
| BMI  Normal/underweight  Overweight | 102^‡^  65^‡^ | 58.8  75.4 | 1.93 (1.08 – 3.43) | 0.03 | 1.38 (0.76 – 2.52) | 0.29 |
| Smoking status  Never smoker  Current/prior smoker | 126^‡^  124^‡^ | 54.8  46.8 | 0.80 (0.57 – 1.15) | 0.24 | 0.80 (0.44 – 1.44) | 0.46 |
| irAE  Yes  No | 109  151 | 69.7  36.4 | 0.34 (0.23 – 0.50) | <0.001 | 0.55 (0.30 – 0.99) | 0.05 |
| Prior systemic therapies  0  ≥1 | 141  119 | 61.7  37.0 | 0.47 (0.33 - 0.67) | <0.001 | 0.66 (0.34 – 1.28) | 0.22 |
| Bone metastases  Yes  No | 39  221 | 38.5  52.5 | 1.58 (1.01 – 2.46) | 0.02 | 1.36 (0.71 – 2.62) | 0.35 |
| Liver metastases  Yes  No | 75  185 | 36.0  56.2 | 1.81 (1.26 – 2.58) | 0.001 | 1.48 (0.82 – 2.66) | 0.19 |
| Lung metastases  Yes  No | 166  94 | 49.4  52.1 | 1.06 (0.74 – 1.52) | 0.76 | 1.46 (0.76 – 2.83) | 0.26 |
| LN metastases  Yes  No | 224  36 | 47.8  66.7 | 1.58 (1.01 – 2.46) | 0.04 | 1.90 (0.65 – 5.62) | 0.24 |
| Brain metastases  Yes  No | 62  198 | 46.8  51.5 | 1.21 (0.82 – 1.80) | 0.34 | 1.41 (0.76 – 2.62) | 0.27 |

n: Number; OS: Overall survival; HR: Hazard ratio; CI: Confidence interval; ECOG PS: European Cooperative Oncology Group Performance Status; BMI: Body Mass Index; irAE: Immune-related adverse event; LN: Lymph nodes

^†^ Excludes patients with mesothelioma (n=4)

^‡^ Data missing for some patients

**Supplementary Table 5: Univariate and multivariate analysis with odds ratio for 5-year Overall Survival**^†^

| Variable | Univariate | | | Multivariable | | |
| --- | --- | --- | --- | --- | --- | --- |
|  | **OR** | **95% CI** | **P value** | **OR** | **95% CI** | **P value** |
| Sex, male vs. female | 0.83 | 0.49 – 1.41 | 0.50 | 0.97 | 0.45 – 2.07 | 0.93 |
| Age, <65y vs. ≥65y | 1.67 | 1.01 – 2.75 | 0.05 | 1.65 | 0.80 – 3.39 | 0.17 |
| ECOG PS, 0 vs. ≥1 | 5.53 | 2.90 – 10.53 | <0.001 | 3.86 | 1.62 – 9.22 | <0.01 |
| irAE, yes vs. no | 3.08 | 1.83 – 5.16 | <0.001 | 1.62 | 1.05 – 3.50 | 0.05 |
| Prior therapies, 0, ≥ 1 | 2.57 | 1.53 – 4.32 | <0.001 | 1.47 | 0.66 – 3.30 | 0.35 |
| BMI  Normal vs. overweight^†^ | 0.66 | 0.35 – 1.23 | 0.19 | 1.10 | 0.51 – 2.34 | 0.82 |
| Smoking,  Never smoker vs. Smoker^†^ | 1.27 | 0.77 – 2.11 | 0.35 | 1.78 | 0.84 – 3.72 | 0.13 |
| Bone metastases,  yes vs. no | 0.28 | 0.12 – 0.67 | <0.01 | 0.26 | 0.09 – 0.79 | 0.02 |
| Liver metastases,  yes vs. no | 0.41 | 0.23 – 0.74 | <0.01 | 0.32 | 0.13 – 0.79 | 0.01 |
| Lung Metastases,  yes vs. no | 1.17 | 0.70 – 1.97 | 0.56 | 1.36 | 0.63 – 2.97 | 0.44 |
| Lymph node metastases,  yes vs. no | 0.47 | 0.23 – 0.96 | 0.04 | 0.38 | 0.12 – 1.24 | 0.11 |
| Brain Metastases,  yes vs. no | 0.95 | 0.53 – 1.71 | 0.87 | 0.75 | 0.31 – 1.80 | 0.52 |

n: Number; OR: Odds ratio; CI: Confidence interval; ECOG PS: European Cooperative Oncology Group Performance Status; BMI: Body Mass Index; irAE: Immune-related adverse event; LN: Lymph nodes

^†^ Excludes patients with mesothelioma (n=4)

**Supplementary Table 6: Univariate and multivariate analysis with hazard ratio for 5-year Overall Survival in the melanoma cohort**

|  |  |  | Univariate Analysis | | Multivariate Analysis | |
| --- | --- | --- | --- | --- | --- | --- |
| Variable | **Patients**  **(n=202**) | **5 year OS, %** | **Hazard Ratio (95% CI)** | **P value** | **Hazard Ratio (95% CI)** | **P value** |
| Age, y  < 65  ≥ 65 | 98  104 | 48.0  36.5 | 0.69 (0.48 – 1.01) | 0.05 | 0.56 (0.29 – 1.06) | 0.07 |
| Sex  Male  Female | 137  65 | 38.7  49.2 | 1.35 (0.90 – 2.03) | 0.15 | 1.15 (0.58 – 2.30) | 0.68 |
| ECOG PS  0  ≥1 | 142  60 | 52.1  18.3 | 0.35 (0.24 – 0.51) | <0.001 | 0.40 (0.18 – 0.89) | 0.03 |
| BMI  N/underweight  Overweight | 67^†^  47^†^ | 59.7  61.7 | 1.12 (0.61 – 2.02) | 0.72 | 0.74 (0.38 – 1.46) | 0.40 |
| Smoking status  Never smoker  Current/prior smoker | 108^†^  84^†^ | 44.4  40.5 | 0.90 (0.62 – 1.31) | 0.58 | 0.76 (0.40-1.47) | 0.42 |
| BRAF  Negative  Positive | 163  39 | 47.5  20.5 | 0.45 (0.30 – 0.68) | <0.001 | 0.49 (0.14 – 1.74) | 0.27 |
| irAE  Yes  No | 91  111 | 55  28.7 | 0.39 (0.26 – 0.58) | <0.001 | 0.46 (0.24 – 0.91) | 0.03 |
| Prior systemic therapies  0  ≥1 | 125  77 | 49.6  29.6 | 0.55 (0.38 – 0.80) | <0.01 | 1.17 (0.43 – 3.20) | 0.76 |
| Bone metastases  Yes  No | 24  178 | 20.8  44.9 | 1.72 (1.04 – 2.84) | 0.04 | 1.84 (0.83 – 4.11) | 0.14 |
| Liver metastases  Yes  No | 63  139 | 28.6  48.2 | 1.79 (1.23 – 2.61) | <0.01 | 2.74 (1.33 – 5.61) | <0.01 |
| Lung metastases  Yes  No | 116  86 | 45.7  37.2 | 0.83 (0.58 – 1.20) | 0.33 | 0.93 (0.49 – 1.78) | 0.83 |
| LN metastases  Yes  No | 168  34 | 39.3  55.9 | 1.55 (1.90 – 2.67) | 0.12 | 2.35 (0.84 – 6.58) | 0.10 |
| Brain metastases  Yes  No | 54  148 | 42.6  41.9 | 1.07 (0.71 – 1.62) | 0.74 | 1.24 (0.54 -2.84) | 0.62 |

n: Number; OS: Overall survival; HR: Hazard ratio; CI: Confidence interval; ECOG PS: European Cooperative Oncology Group Performance Status; BMI: Body mass index; irAE: Immune-related adverse events; LN: Lymph nodes

^†^ Data missing for some patients

**Supplementary Table 7: Immune-related adverse events by affected organ and grade**

|  | irAE (n) ^†^ | irAE grade 3 or higher (n) |
| --- | --- | --- |
| Thyroiditis | 28 | 2 |
| Hepatoxicity | 12 | 6 |
| ITP | 1 | 1 |
| Pneumonitis | 11 | 4 |
| Hypophysitis | 20 | 2 |
| Pancreatitis | 3 | 2 |
| Arthritis | 15 | 3 |
| Rash | 29 | 6 |
| Colitis | 18 | 7 |
| Diabetes Mellitus | 1 | 0 |
| Myocarditis | 1 | 1 |
| Nephritis | 5 | 2 |
| Transverse Myelitis | 1 | 1 |

irAE: Immune-related adverse events; n: Number; ITP: Immune thrombocytopenia

^†^Some patients had more than one type of irAE
